# Structural changes and adaptative evolutionary constraints in FLOWERING LOCUS T and TERMINAL FLOWER1-like genes of flowering plants

**DOI:** 10.3389/fgene.2022.954015

**Published:** 2022-09-29

**Authors:** Deivid Almeida de Jesus, Darlisson Mesquista Batista, Elton Figueira Monteiro, Shayla Salzman, Lucas Miguel Carvalho, Kauê Santana, Thiago André

**Affiliations:** ^1^ Institute of Biology Genetics Graduate Program, Federal University of Rio de Janeiro Rio de Janeiro, Rio de Janeiro, Brazil; ^2^ Programa de Pós-Graduação em Biodiversidade, Universidade Federal do Oeste do Pará Santarém, Pará, Santarém, Brazil; ^3^ School of Integrative Plant Sciences. Section of Plant Biology. Cornell University Ithaca, New York, NY, United States; ^4^ Center for Computing in Engineering and Sciences, State University of Campinas. Campinas, São Paulo, Brazil; ^5^ Institute of Biodiversity, Federal University of Western Pará Santarém Pará, Santarém, Brazil; ^6^ Botany Department, University of Brasília, Brasília, Brazil

**Keywords:** phenology, structural Biology, phylogenetics, protein structure prediction, adaptative evolutionary constraints, natural selection

## Abstract

Regulation of flowering is a crucial event in the evolutionary history of angiosperms. The production of flowers is regulated through the integration of different environmental and endogenous stimuli, many of which involve the activation of different genes in a hierarchical and complex signaling network. The *FLOWERING LOCUS T*/*TERMINAL FLOWER 1* (*FT/TFL1*) gene family is known to regulate important aspects of flowering in plants. To better understand the pivotal events that changed FT and TFL1 functions during the evolution of angiosperms, we reconstructed the ancestral sequences of *FT*/*TFL1-like* genes and predicted protein structures through *in silico* modeling to identify determinant sites that evolved in both proteins and allowed the adaptative diversification in the flowering phenology and developmental processes. In addition, we demonstrate that the occurrence of destabilizing mutations in residues located at the phosphatidylcholine binding sites of FT structure are under positive selection, and some residues of 4^th^ exon are under negative selection, which is compensated by the occurrence of stabilizing mutations in key regions and the P-loop to maintain the overall protein stability. Our results shed light on the evolutionary history of key genes involved in the diversification of angiosperms.

## Article highlights

• FT and TFL1 have mostly been evolving under purifying selection as evidenced by substitutions in the third position of the codons that encode key residues involved with FT/TFL1 function do not alter the encoded amino acids.
• Residues from the P-loop domain of the analyzed FT structures show predominantly high destabilizing mutations which is consistent with constant selective pressure found for this region.
• Protein conformation more than sequence appears to be under strong selective pressure in that amino acid substitutions that would have resulted in structural changes in over evolutionary time show matching and stabilizing mutations.
• The appearance of destabilizing mutations in residues of phosphatidylcholine binding sites are under negative selection, while some residues of the 4th exon are under positive selection, these structural changes are compensated by the occurrence of stabilizing mutations in key regions to maintain the overall stability of the protein.
• The presence of destabilizing mutations and negative selective pressures in residues located at the phosphatidylcholine binding site involved with H-bond formation indicate their structural role in maintaining the overall stability of FT structure.

## Introduction

Flowering is a major event in the angiosperms life cycle because it allows sexual reproduction (Jin et al., 2020). The production of reproductive meristems and flower organs is regulated through the integration of environmental and endogenous stimulus (Ahn et al., 2006a; Pin and Nilsson, 2012) involving the activation of different genes and a complex and hierarchical signaling network. The *FLOWERING LOCUS T* (*FT*) and *TERMINAL FLOWER 1* (*TFL1*) gene family are known to regulate important aspects of growth and flowering in plants (Pin and Nilsson, 2012; Jin et al., 2020). FT proteins are key regulators activated by the transcription factor CONSTANS (CO) and are involved with the control of multiple flowering pathways in angiosperms (Kobayashi et al., 1999; Kim et al., 2008; Ogiso-Tanaka et al., 2013; Fan et al., 2020). When activated, the FT interacts with the FD, a basic leucine zipper domain transcription factor (bZIP), and induces the expression of *APETALA1* (*AP1*) and *SUPPRESSOR OF OVEREXPRESSION OF CONSTANS1* (*SOC1*) genes, leading to flower development (Wigge, 2005; Collani et al., 2019; Zhu et al., 2020). Contrasting FT activity, TFL1 proteins are known to repress flowering by inhibiting the expression of key flowering pathway genes (Kardailsky et al., 1999; Lifschitz et al., 2006). Besides flowering regulation, FT/TFL1-like proteins have also been identified as regulatory factors in a wide range of developmental processes in plants that includes seed germination (Xi et al., 2010), stomatal opening (Kinoshita et al., 2011), response to extended cold winter temperatures (Pin et al., 2010), control of the lateral shoot development (Hiraoka et al., 2013), and formation of storage organs (Navarro et al., 2011).

FT and TFL1 proteins are homologs to phosphatidylethanolamine-binding proteins (PEBPs) which are all involved in the signaling pathways that control differentiation of stem apical meristem (Bernier and Périlleux, 2005a; Liu et al., 2016). Genes reported belonging to PEBPs superfamily include *CENTRORADIALIS* (*CEN*) (Banfield and Brady, 2000), *TWIN SISTER OF FT* (*TSF*) (Yamaguchi et al., 2005a), *BROTHER OF FT AND TFL1* (*BFT*) (Yoo et al., 2010), and *MOTHER OF FT AND TFL1* (*MFT*) (Yu et al., 2019), and others (Bernier and Périlleux, 2005b). Regarding the evolution of these genetic regulators, phylogenetic analyses revealed that these PEBPs-like genes are grouped in three main clades: *FT*-like, *TFL1*-like, and *MFT*-like genes (Chardon and Damerval, 2005; Carmona et al., 2007; Zheng et al., 2016). Similarly, gymnosperms possess two groups: *MFT*-like and a group that occupies an intermediate position between the *FT*- and *TFL1*-like (*FT/TFL1*-like) genes (Karlgren et al., 2011a). Recently studies have demonstrated that *FT/TFL1*-like sequences were present in gymnosperms lineages in duplicates, which could have occurred even prior to the emergence of seed plants (Liu et al., 2016). Genomic analyses revealed that gene duplications played an important role in the diversification of gene function in angiosperms, which were essential for adaptative evolution (Soltis et al., 2015). Different studies have reported that *MFT* genes are ancestral to *FT* and *TFL1*, and the origin of these orthologue genes is related to the occurrence of duplication events in the evolutionary history of angiosperms (Hedman et al., 2009; Karlgren et al., 2011a; Wickland and Hanzawa, 2015).

The FT and TFL1 of *Arabidopsis thaliana* exhibit conserved structures (Ahn et al., 2006a; Pin and Nilsson, 2012) with small tractable changes differentiating them. Studies have demonstrated that mutations in four key residues, Glu109, Trp138, Gln140, and Asn152 could transform the activator function of FT into the suppressor activity of TFL1 (Hanzawa et al., 2005; Ho and Weigel, 2014). In addition, an external loop region of 14 residues in FT named the P-loop (*A. thaliana* position 139–152), confers an antagonistic activity to the floral regulators (Ahn et al., 2006a; Pin and Nilsson, 2012). To better understand the pivotal events that changed FT and TFL1 functions during the evolution of flowering plants and the structural role of residue sites in both proteins throughout the diversification of angiosperms, we reconstructed ancestral sequences of the *FT/TFL1* genes, predicted the corresponding protein structures and performed structural mutational analyzes utilizing the genetic model of *A. thaliana*. Our results shed light on the role of natural selection in the adaptive evolution of flowering proteins in angiosperms and can help in the improvement of crops with economic interest and for the flower and fruit industries.

## Materials and methods

### Phylogenetic analysis and ancestral sequence inference

First, we performed a sequence alignment search to find *FT/TFL1*-like homologs (Supplementary Information S1) using as a start point the coding DNA sequences (CDSs) of *FT* and *TFL1* from *Arabidopsis thaliana* (GeneBank accession codes: *FT*: NM_105222.3; *TFL1*: NM_120465.3). The similarity sequence search was performed through the BLASTn tool using the GenBank database (Benson et al., 2017). Sequences with the highest identities, percent similar identities above 82%, were included in further analysis. In addition, sequences from different species were included in our analysis based on previous findings (Ahn et al., 2006b; Ho and Weigel, 2014).

To infer gene phylogenetic trees, we used a total of 103 coding sequences of *FT* (100 sequences from angiosperms, belonging to monocots, eudicots, asterids, and brassicales groups; and 3 sequences from gymnosperms); and 82 sequences of *TFL1* (78 sequences from angiosperms belonging to monocots and eudicots; and 4 sequences from gymnosperms). In both phylogenetic inferences, gymnosperm sequences of *FT* and *TFL1* were used as outgroups (Supplementary Information S1). Multiple sequence alignments were performed in MUSCLE (Edgar, 2004) as implemented in MEGA 7 (Kumar et al., 2016). These alignments were further manually checked and edited, mainly to maintain reading frames. The jModel Test 2 program was used to select the nucleotide model with the best Bayesian information criterion (BIC) score (Darriba et al., 2012). We used MEGA7 (Kumar et al., 2016) for ancestral sequence reconstruction (ASR) using the Maximum Likelihood method to estimate the ancestral state of each node in a phylogeny where the state is chosen to be the one that maximizes the probability of the sequence data based on the selected evolutionary model of nucleotide or amino acid substitutions. Bayesian phylogenetic inference was executed in BEAST version 1.8.4 (Drummond et al., 2012). A relaxed clock with an uncorrelated lognormal model of rate variation was used and the Yule speciation process for branching rates was selected. Seventeen fossil-based times to the most recent common ancestor (mrca) calibrations were used based on published data. Calibration dates and associated citations can be found in Supplementary Information S2. A CTMC rate prior was selected and no monophyletic prior assignment was made. Markov chain Monte Carlo simulations were run for 5 × 10^7^ generations and sampled every 1 × 10^3^. These analyses were performed in CIPRES Science Gateway server v3.3 (Miller et al., 2010). To analyze the continuous parameter values sampled from the Bayesian chains, we assessed the convergence of the models across independent runs by analyzing plots of the marginal later distributions in Tracer (version 1.7.1) (Rambaut et al., 2018). To ensure high effective sample size (ESS) values, we considered a value above or equal to 200 (ESS ≥200). Tracer was also used to assess burn-in levels and a maximum clade-credibility tree was obtained from the later sample of trees using TreeAnnotator v. 1.7.1 (Drummond et al., 2012).

### *In silico* prediction of FT and TFL1 structures

Putative structures of FT/TFL1 proteins were generated through comparative modeling in Modeller program version 9.19 (Fiser and Šali, 2003), which uses the satisfaction of the spatial restraints method. The crystallographic structures of FT (PDB code: 1WKP, chain A; resolution: 2.6Å) and TFL1 (PDB code: 1WKO, chain A; resolution: 2.6Å) from *A. thaliana* were used as templates. To predict structures, we performed a pairwise sequence alignment using the BLOSUM62 (20 × 20) matrix. The models of both proteins were optimized at the atomic level using the random parameter in the range [0.400] and resistance [0,20] e in the ModRefiner program (Xu and Zhang, 2011) and then, minimized by 1,000 cycles of conjugated gradient and 1,000 cycles of steepest-descent algorithms in the Amber16 package (Salomon-Ferrer et al., 2013). The modeled protein structures were validated by the stereochemical quality using the Ramachandran plot obtained in Procheck program version 3.5.4 (Laskowski et al., 1993) and the energetic profile obtained by the Qmean plot (Benkert et al., 2009), both using default parameters. Moreover, structural alignment and RMSD-Cα values were used to evaluate the conservation of the modeled FT and TFL1 structures with the selected templates. Finally, to analyze the surface potentials of the FT and TFL1 regions involved in their molecular activity, we obtained the Poisson-Boltzmann electrostatic potential map using the PDB2PQR server (Dolinsky et al., 2004) using the parameters of the Amber forcefield.

### Molecular dynamics simulation

To analyze structural changes in FT and TFL1, molecular dynamics (MD) simulations were performed in the Amber16 package (Case et al., 2005). The all-atom forcefield Amberff14SB was used to parameterize the protein structures. The proteins were solvated in a truncated octahedral water-box with the explicit solvation model TIP3P (Jorgensen et al., 1996). We used a distance of 10 Å between the cell wall and the solvated atoms of the system, and a distance of 0.8 Å between water molecules and the solute. Counter-ions Cl^−^ were also added to neutralize the analyzed systems. Initially, all hydrogen atoms of the system were minimized for 3,000 cycles of the steepest-descent (Wiberg, 1965) and 3,000 cycles of the conjugate gradient algorithm (Hestenes and Stiefel, 1952). Water and ions were minimized for 2,000 cycles of the steepest-descent and 3,000 cycles of the conjugate gradient. Then, the whole system was minimized using 2,000 cycles of the steepest descent and 3,000 cycles of the conjugate gradient; and we performed seven repetitions, with progressive relaxing of restraints. After completion of minimization, the system was gradually heated to increase the temperature to 300 K during 4.25 ns of equilibration. Then, the MD was performed with the isobaric-isothermal ensemble with a total time of 20 ns. The temperature was maintained using the Langevin thermostat and the SHAKE algorithm was applied to all hydrogens of the system, which allowed us to use integration cycles of 2.0 fs and the constant isotropic pressure was maintained at 1 bar by using the Berendsen barostat. A cutoff of 10 Å was used for the minimum image convention. The values of the RMSD and RMSF, based on the heavy atoms of the protein backbone were used to determine the conformational changes over the MD simulations.

### Mutational analysis of protein structures

To analyze the effects of mutations in the coding sequences on the structural analysis of the proteins, alanine scanning was performed using the FoldX program (Guerois et al., 2002). FoldX uses a linear combination of different empirical terms to calculate free energy (ΔG). Empirical terms include Coulomb terms for electrostatic interactions, van der Waals terms, hydrophobic and solvation forces, hydrogen bonds, and so on. The results of mutational analyses (∆∆G_fold_) are expressed by the difference between the free energy of the wild-type (ΔG_wt_) and the mutant (ΔG_mut_) structures (kcal.mol^−1^) according to Eq. 1:

$$\Delta \Delta G_{fold}=\Delta Gfold,wt-\Delta G_{fold,mut}$$ (1)

Where ∆G_fold_,_wt_ is the free energy variation of wild-type structure and ΔG_fold_,_mut_ is the variation of the mutant structure. If ∆∆G < 0, the mutation was considered stabilizing, and if ∆∆G>0, the mutation was considered structurally destabilizing (Morrison & Weiss, 2001). The mutations were classified in five different categories, depending on the implications to the stability of protein structure: highly stabilizing (ΔΔG < −1.84 kcal mol^−1^); slightly stabilizing (−1.84 kcal mol^−1^ ≤ ΔΔG < −0.46 kcal mol^−1^); neutral (−0.46 kcal mol^−1^ < ΔΔG ≤ +0.46 kcal mol^−1^); slightly destabilizing (+0.46 kcal mol^−1^ < ΔΔG ≤ +1.84 kcal mol^−1^); highly destabilizing (ΔΔG > +1.84 kcal mol^−1^).

### Calculations of dN/dS rates

To identify residues under natural selection, i.e., neutral, positive, or negative selections, we calculated the non-synonymous mutation (dN) and the synonymous mutation (dS) rates using the clade and codon models available in the EasyCodeML program (Gao et al., 2019), which implements the clade and codon-based models of CodeML. The clade model was based in the model C (CmC) to estimate a separate ω ratio for each clade, and it was compared against a null model 2a_rel (M2a_rel), in which ω is fixed among the analyzed clades (Anisimova and Kosiol, 2007; Weadick and Chang, 2012). FT’s ω values were obtained for the most recent common ancestor of five major clades: angiosperms, monocots, eudicots, asterids, and brassicales TFL1’s ω values were obtained for angiosperms, monocots, eudicots, brassicales, and non-brassicales. The ω values were validated by likelihood scores.

## Results and discussion

Our results shed a light on the evolutionary history and structural importance of *FT/TFL1*-like genes. Using fossil records, we calibrated our phylogenetic trees, providing more accurate indications of the divergence of both genes during the evolution of angiosperms. We inferred the ancestral sequences and predicted ancestral structures of FT and TFL1 for the major clades of flowering plants. Our results allow us to identify the structural changes of proteins crucial to the flowering process throughout the evolutionary history of angiosperms, such as the electrostatic potential map of the phosphatidylcholine site, as well as, the structural implications of mutations at key residues involved with the molecular function of both FT and TFL1 proteins.

### Reconstruction of evolutionary history of *FT* and *TFL1* in angiosperms

In the present study, when species presented two or more copies for the same gene (interspecies paralogs) within the *FT* group or in *TFL1*, we used both copies in our analyses as previous studies highlighted the impact of ignoring paralogs in generating bias in the prediction of protein function (Stamboulian et al., 2020). TIM2ef + I + G was selected as the most appropriate evolutionary model for the *FT* and *TFL1* sequences. Using a Bayesian relaxed-clock approach, we estimated the phylogeny and the divergence time for *FT* and *TFL1* genes. Our phylogenetic analyses revealed that natural selection acted differently for both genes, thus they did not show the same evolutionary divergence found in angiosperms (Flagel and Wendel, 2009). These results corroborate previous phylogenetic trees obtained for both genes (Wang et al., 2017).

**FIGURE 1:**
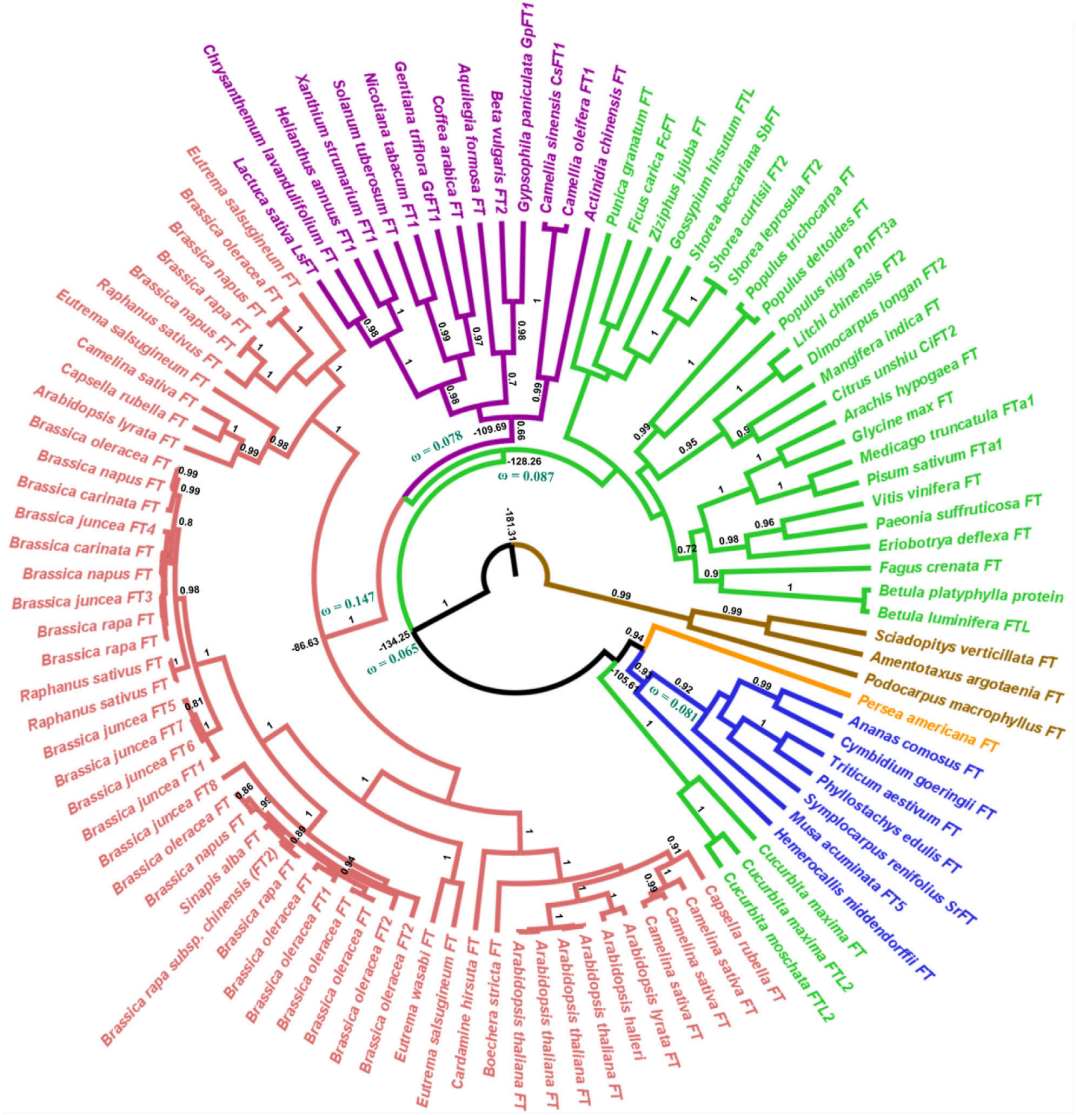
Bayesian phylogeny of angiosperm FT sequences. The major branches are indicated by posterior probability. Molecular dating and the dN/dS ratios represented by the ω value are observed in the major clades. Colors indicate the clades, red = brassicales, purple = asterids, green = eudicots, blue = monocots, orange = Magnoliidae, brown = external group. The brassicales, asterids, and eudicots clades (red, purple, and green) form the ancestor of eudicots.

**FIGURE 2:**
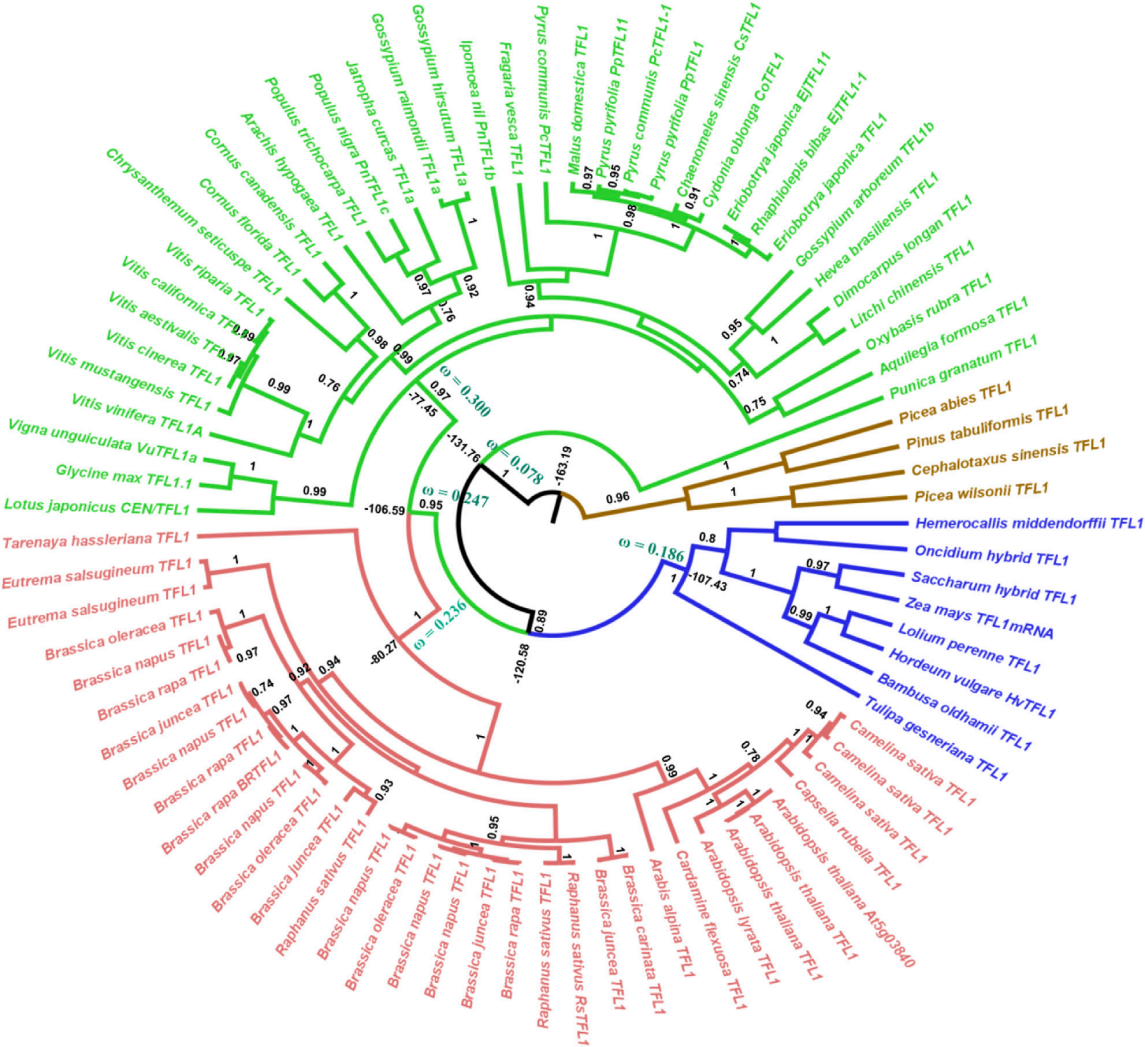
Bayesian phylogeny of angiosperm TFL1 sequences. The major branches are indicated by posterior probability. Molecular dating and the dN/dS ratios represented by the ω value are observed in the major clades. Colors indicate the clades, red = brassicales, green = non-brassicales, blue = monocots, brown = outgroup. The brassicales and non-brassicales clades (red and green) form the ancestor of eudicots.

Our phylogenetic hypothesis for gene evolution demonstrates that *FT*-like genes diverged ca. 181 million years ago and *TFL1*-like genes ca. 163 million years ago. Within the angiosperm clade, the *FT*-like genes diversified approximately 134 million years ago (Figure 1) and the *TFL1*-like genes around 131 million years ago (Figure 2). (Klintenäs et al., 2012a) suggested that *FT* is found exclusively in flowering plants. Here, we used the *FT* and *TFL1* genes from gymnosperms solely to root the phylogenetic tree. The choice of different species was due to the absence of sequences of both genes from the same species available in public databases. However, it has been evidenced in previous studies that gymnosperms carry one or two copies of the *FT/TFL1* genes, that represent a clade that is sibling or ancestor of the *FT/TFL1* genes found in angiosperms (Karlgren et al., 2011a; Klintenäs et al., 2012a).

In the angiosperm clade, we recovered four monophyletic groups of *FT*-like sequences: monocots (posterior probability = 0.93), eudicots (0.90), asterids (0.66), and brassicales (1.0). Regarding the *TFL1* sequences, we also recovered distinct clades for the monocots (1.0), eudicots (0.95), brassicales (1.0), and an unresolved group which we named non-brassicales (0.97) (Figure 1). The estimated time of evolutionary divergence of *FT*-like sequences is approximately 105 million years ago for monocots, 128 for eudicots, 109 for asterids, and 86 for brassicales. Divergence time for *TFL1* sequences was relatively similar, if not slightly earlier. Monocot *TFL1* diverged approximately 107, eudicots 106, brassicales 80, and the non-brassicales group 77 million years ago (Figure 2). It is important to note that a previous study used genetic distances to analyze the divergences of both genes and the authors reported the presence of *FT*-like genes in gymnosperms (Liu et al., 2016). In contrast, our study used fossil records to estimate the divergences between the main groups of *FT* and *TFL1* genes, which may contribute to more accurate findings regarding the divergence of these genes in plant evolution. Herein, we showed that both FT and TFL1 genes had similar divergence times.

We conjecture that differences between the phylogenetic trees of *FT/TFL1*-like genes and the well-known angiosperm phylogeny could be caused by divergent evolution related to the pleiotropic effects exercised by the *FT/TFL1*-like genes in angiosperms, which influence a wide range of developmental stages of plants, such as flowering, seed storage, and stomatal opening (Yamaguchi et al., 2005b; Lifschitz et al., 2006; Xi et al., 2010; Pin and Nilsson, 2012). Pleiotropy has a functional diversity regulating different phenotypic characteristics, thus influencing the natural selection pressures leading to the appearance or elimination of new characteristics (Auge et al., 2019).

We also noted that the sequence of *Aquilegia* sp. Remained in the Asterids clade and this result could be related due to the coalescence evolution of this gene in relation to the taxonomic group (Gatesy et al., 2019). Furthermore, it is important to highlight that the phylogenetic relationships of the *FT* and *TFL1* genes from angiosperms and the basal group of angiosperms have been well reported in previous studies (Karlgren et al., 2011b; Klintenäs et al., 2012a). These studies have demonstrated that the *FT* and *TFL1* genes from these groups do not imply the formation of independent clades which is similar to our reported phylogenetic results. In addition, our phylogenetic analysis shows the formation of well-supported clades for the main monophyletic groups of angiosperms, thus indicating that the natural selection acts to preserve the *FT/TFL1* functions in each clade during the evolutionary divergence of these genes. Indeed, a prior study of dN/dS rates in duplicated *FT/TFL1*-like genes showed that they suffer negative pressures (Mackenzie et al., 2019a).

### Inference of the Ancestral Sequences of *FT* and *TFL1*

Based on the phylogenetic trees, we selected five ancestral *FT*- and *TFL1*-like sequences, which are representative of five clades with satisfactory posterior support: monocots, eudicots, asterids (*FT*), brassicales, and non-brassicales (*TFL1*). The ancestral sequence reconstruction used here, refers to the reconstruction of the ancestral state of each node in a phylogenetic tree. The state is chosen to maximize the probability of the sequence data given under the nucleotide evolution likelihood probability model. The result obtained is a consensus nucleotide sequence used as input for molecular modeling and in the natural selection analysis software. Moreover, we analyzed the current representatives of protein structures (FT and TFL1) in *A. thaliana* (reference structure).

**FIGURE 3:**
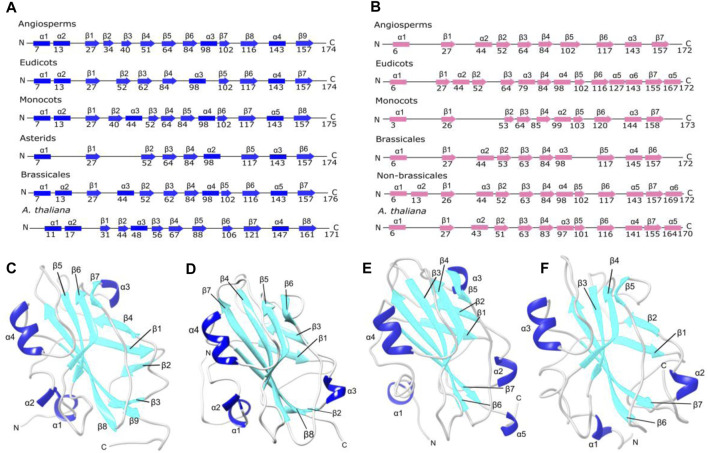
Protein secondary and tertiary structures of FT and TFL1 **(A)** Secondary structures of inferred ancestral and *A. thaliana* FT. **(B)** Secondary structures of ancestral and *A. thaliana* TFL1 **(C)** Tertiary structure of the inferred ancestral of angiosperm clade FT. **(D)** Tertiary structure of *A. thaliana* FT **(E)** Tertiary structure of *A. thaliana* TFL1. **(F)** Tertiary structure of the inferred ancestral of angiosperm clade TFL1.

Structurally, FT shows seven β-strands and four α-helices, except the ancestral structure of the asterids clade that exhibits three α-helices and six β-strands, FT structure from ancestral angiosperms shows four α-helices and nine β-strands (Figure 3, panel A and C). In contrast, the TFL1 structures contain predominantly three α-helices and seven β-strands (Figures 3B–F).

**FIGURE 4:**
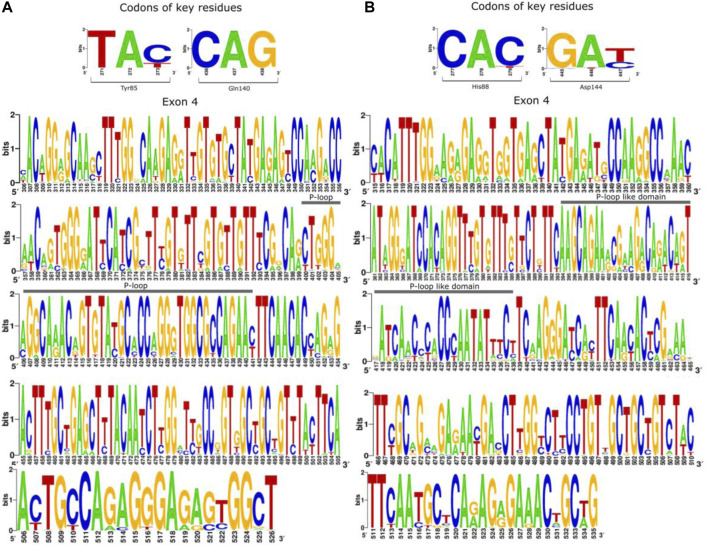
The 4th exon that encodes the P-loop domain in *FT* structure is well-conserved over the evolutionary history of angiosperms. Schematic overview of ancestral codon sequences of the 4th exon showing the conserved regions of *FT* **(A)** and *TFL1* **(B)**. The height of each nucleotide within the pictogram indicates their relative frequency in the analyzed position. It is important to note that the numbering of the residues from the P-loop is different in the angiosperm sequences, however, its position remains the same for all analyzed sequences.

Studies have demonstrated that a short loop segment located between the residues 128 to 145 (P-loop domain) is well conserved across plant families and is the major determinant of FT activity (Pin et al., 2010; Pin and Nilsson, 2012). Our reconstruction of ancestral angiosperm FT found both the highly conserved P-loop domain (ancestral sequences: Leu127 to Asn142; *A. thaliana:* Leu131 to Asn146) as well as the key residues Tyr85 and Gln140 (Figure 4, panel A). In contrast, the homolog region of P-loop from TFL1 ancestral sequences is less conserved showing more substitutions through the evolutionary history of angiosperms, which suggests the non-functionality of these regions in flowering repression (Figure 4B). However, the key-residue His88 was found to be conserved in all analyzed ancestral sequences of angiosperms.

A previous study demonstrated that the flowering time in sugar beet (*Beta vulgaris* ssp. *Vulgaris*; Eudicots clade) is controlled by the combined activity of two paralogs, which have an antagonistic function: *BvFT1* and *BvFT2* (Pin et al., 2010). The P-loop domain of BvFT1 and BvFT2 show substitutions in the residues Tyr134Asn and Trp138Gln (*B. vulgaris* spp*. Vulgaris* numbering), which are involved in flowering repression (Pin and Nilsson, 2012). In the present study, we noted a new residue position for the analyzed ancestral sequences of the P-loop domain when compared with the *A. thaliana* sequence. Within TFL1, the residues Pro134 and Arg138 found in *A. thaliana* remained conserved in the analyzed ancestral sequences and a substitution in the homolog region of the P-loop domain was found at Pro134Asn in the ancestral sequence of monocots. A possible explanation for this change occurring only in monocots is based on the findings by (Ospina-Zapata et al., 2020) in which monocots show greater recruitment of homologous copies of *FT* that play a role in repressing flowering, as it is attributed to *TFL1*. In contrast, the *TFL1-*like sequences are kept in a few copies, and in some species, they can be lost or maintained with neutral mutations (see section mutational analysis) to allow mutations like the ones presented here. Analysis of the transcriptome of different species of monocots combined with mutagenesis approaches may provide better evidence.

Ho and Weigel (2014) identified that the position of the residue 134 and 138 in the FT structure of *A. thaliana* are located at the external P-loop domain and easily accessible to the surface of the phosphatidylcholine (PC) binding site, thus indicating interaction with other molecules (Ho and Weigel, 2014). It is therefore expected that purifying selection acts to conserve this structure over evolutionary time. Analyzing the nucleotide frequency that encodes the key residues for FT/TLF1 function, as well as the C-terminal region of both proteins, we observed that substitution in the third position of codons that encode key residues of FT (Tyr85 and Gln140) and TFL1 (His88 and Asp144) involved with molecular function does not alter the encoded amino acids (Supplementary Information S3). This is in accordance with the dN/dS rates found for this region and indicates the existence of purifying selection on these codon sites for both genes. We observed negative selection for the homologous P-loop region of the TFL1 domain, even though variations in the frequency of some codons were observed (Figure 4B).

### Modeled structures of ancestral FT and TFL1 and *A. thaliana*

The modeled structures of the FT/TFL1 proteins exhibited >90% of residues in favorable regions of Ramachandran plot (sum of residues in most favorable and permitted regions, Supplementary Information S4, S5), and a satisfactory energetic profile as exhibited by the local quality estimation of Qmean (Supplementary Information S6, S7), which indicate reliable structures. We performed a short MD simulation to reach the final conformation of the modeled proteins structures. This computational procedure was necessary to correct some stereochemical inconsistences obtained from the comparative modeling of the FT and TFL1 structures. In addition, the RMSD plots obtained over the MD simulation showed that modeled structures reached a stable conformation after 18ns (Supplementary Information S8).

**FIGURE 5:**
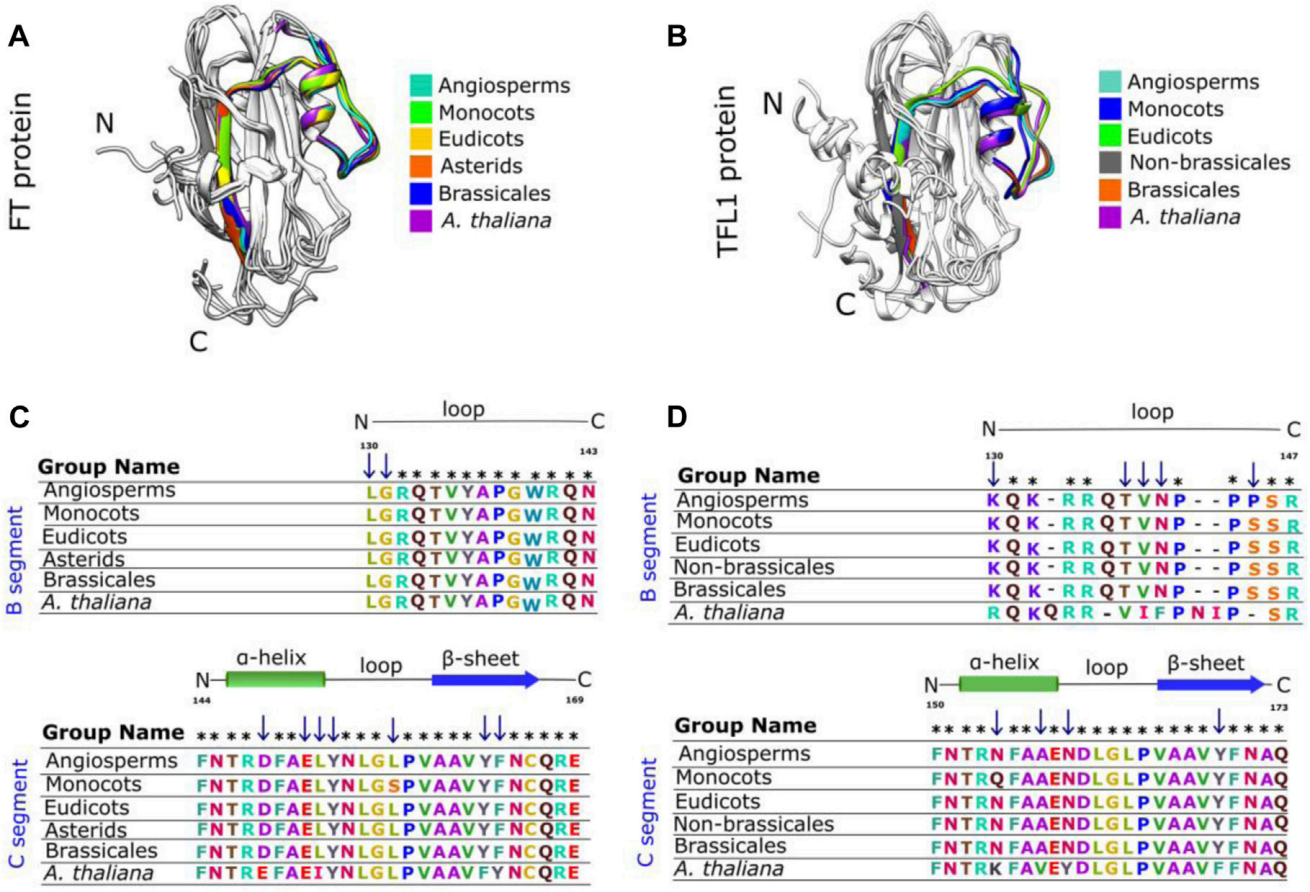
The segment C of FT and TFL1 protein structures are conserved across extant and reconstructed ancestral sequences even with amino acid substitutions. Structural alignment of the segments C from the ancestral FT **(A)** and TFL1 **(B)** sequences, which are coded by the fourth exon. Sequence alignment of the FT **(C)** and TFL1 **(D)** ancestors. Asterisks indicate matches in the alignment sequences, and the blue arrows indicate the residues substitutions.

Previous studies have demonstrated that the segment C located at the C-terminal region of FT/TFL1 structures is crucial for the molecular function of both proteins (Ahn et al., 2006a; Hedman et al., 2009). Based on this assumption, we performed a comparison using the structural alignment of the C-terminal region of the ancestors of FT and TFL1 following the divergence time of the phylogenies (Figures 1, 2). We observed that the ancestral protein of FT in eudicots, which was the first ancestral state to diverge from the main groups of flowering plants, showed high conservation regarding the segment C when compared with its ancestor in angiosperms (Table 1). The ancestor of monocots showed a slightly more divergent RMSD-Cα value between the analyzed FT structures. The FT protein of asterids is an ancestor that evolved within the lineage of eudicots and it also showed high conservation for the same segment. In contrast to eudicots ancestral structure, the brassicales structure, showed a higher RMSDs-Cα value when aligned a eudicots. Finally, the analysis of the FT structure of *A. thaliana*, revealed that the segment C evolved experiencing few mutations which could explain its conserved function over time. The structural alignment of FT showed RMSDs-Cα values ​​≤ 1,153 Å. In contrast, the TFL1 showed RMSDs-Cα values ​​≥ 1,513 Å (Table 1). The segment C of the FT ancestral protein of monocots, when compared to the structure of the ancestral angiosperms, showed to be more conserved than its phylogenetic most evolutionary related group, the eudicots. Comparing the ancestors derived from the lineage of eudicots, brassicales had the lowest RMSDs-Cα value and therefore, the most conserved segment C among all the analyzed ancestors, while non-brasicales showed the highest RMSDs-Cα value, and thus the higher structural variation, followed by eudicots and *A. thaliana* FT structures, respectively. We hypothesize that both proteins evolved independently with natural selection maintaining the most important regions, such as the segment C. We also observed that the ancestral structures of FT proteins showed to be conserved regarding its folding (Figure 5A), exhibiting an α-helix, and a β-strand in segment C (Ahn et al., 2006a).

**TABLE 1:** Structural comparison between segments C of modeled ancestral structures of FT and TFL1. RMSD-C_α_ values exhibited in angstroms.

FT-like structures		TFL1-like structures	
Taxonomic groups	RMSD-Cα (Å)	Taxonomic groups	RMSD-Cα (Å)
Angiosperms with Eudicots	0.845	Angiosperms with Monocots	1.618
Angiosperms with Monocots	1.153	Angiosperms with Eudicots	1.927
Eudicots with Asterids	0.870	Eudicots with Brassicales	1.513
Eudicots with Brassicales	1.051	Eudicots with non-Brassicales	2.047
Brassicales with *A. thaliana*	0.869	Eudicots with *A. thaliana*	1.932

The sequence of segment C of FT from *A. thaliana* showed mutations at three residue positions (Asp146Glu, Leu150IIeu, and Phe162Tyr) when compared with the other analyzed ancestral sequences (Figure 5C). Structural conservation was also found in the C-terminal region of TFL1 which is composed of an α-helix and a β-strand (Figure 5B). The structural alignment of segment C showed slight variations in the RMSD-Cα values between the ancestral states analyzed over the evolutionary history of angiosperms, which demonstrated that those structures have been conserved during evolution (Table 1). However, it is interesting to note some mutations in four residues in TFL1 sequences of *A. thaliana* at the positions Asn150Lys, Ala153Val, Asn155Tyr, and Tyr165Phe when compared with the ancestral sequences.

Comparing the modeled structures of the ancestral lineages of angiosperms, monocots, eudicots, asterids, and brassicales clades, we noted that these proteins remained well-conserved, showing only one substitution at the residue Ser155 in the ancestral monocot structure when compared with the other ancestral sequences. In contrast, compared with *A. thaliana*, this position contains a leucine substitution (Figure 5C). Comparing the *in silico* modeled TFL1 ancestral structures, we identified a mutation at the position Gln146Asn in the ancestral sequence of monocots (Figure 5D), we further discuss possible explanations of these changes below in the mutation analysis section. The residues Tyr85 and Gln140 (*A. thaliana* numbering) involved in the repression of FT activity and His88 and Asp144 (*A. thaliana* numbering) involved in the activation of TFL1, remained conserved in all inferred ancestral sequences of angiosperms, which is consistent with the molecular function previously described for these residues (Ahn et al., 2006a; Ho and Weigel, 2014).

**FIGURE 6:**
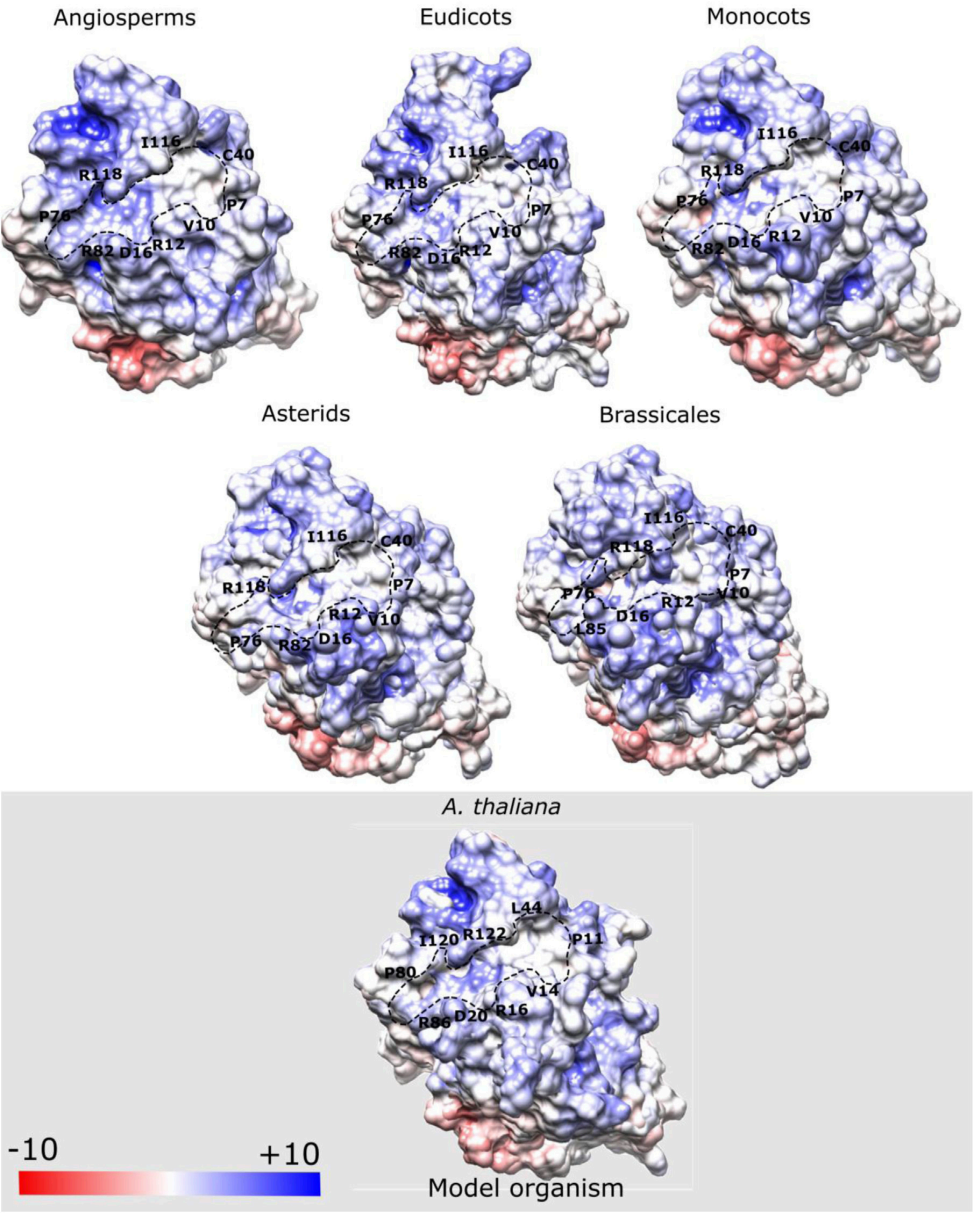
Electrostatic potential map (kBT/e) of phosphatidylcholine (PC) binding site of modeled ancestral FT structures. Some residues located at the PC binding site, such as Pro8, Val11, Arg13, Asp17, Leu41, Pro77, Arg83, Ile117, and Arg119 exhibit a predominantly positive potential. Blue regions indicate the positive potential, white indicates the neutral potential (no charge), and the red regions negative potential. Highlighted residues belong to the binding pocket.

Flowering time in angiosperms is regulated in part by phosphatidylcholine (PC) interaction with FT protein (Nakamura et al., 2014). Therefore, we analyzed the distribution of electrostatic charges (in kBT/e) on the surface of the PC binding site of FT to understand how this region changes across the ancestral structures of the main clades of angiosperms (Figure 6). We observed that the electrostatic surface of the residues located at the PC binding site, such as Pro8, Val11, Arg13, Asp17, Leu41, Pro77, Arg83, Ile117, and Arg119 (*A. thaliana* numbering) exhibited a predominantly positive potential in the ancestral sequences, which is in accordance with previous findings for the FT structure of *A. thaliana* (Nakamura et al., 2019). Some residues considered important for P-loop domain function, such as Glu109 and Gln140 maintained predominantly negative charges which corroborate with the results reported by Ho and Weigel (2014). However, in the present study, we performed a complete analysis of the potential charge of the P-loop domain and found that this domain shows a potential charge conserved in the ancestral and current FT structures.

**FIGURE 7:**
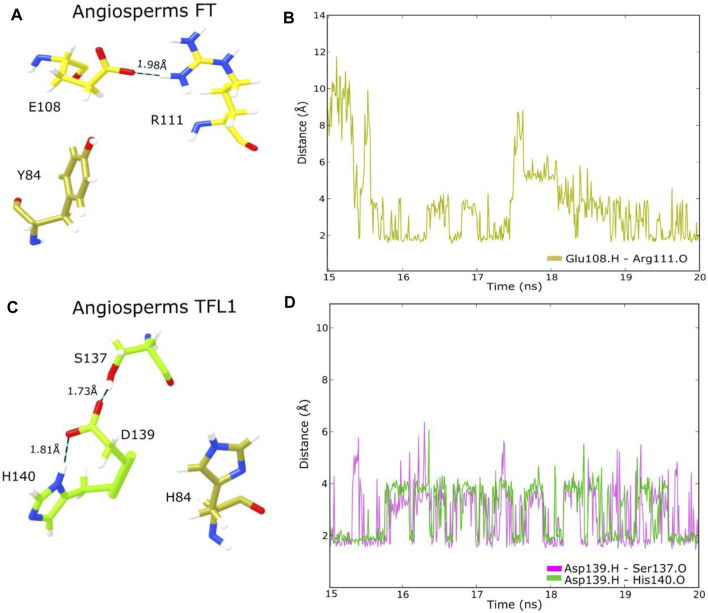
Analysis of H-bond interactions in the ancestral FT and TFL1 structures from angiosperms through MD simulation **(A)** Detailed overview of FT residues involved in H-bond interactions; **(B)** Interatomic H-bond distances verified over 20 ns of MD simulation of FT structure **(C)** Detailed overview of TFL1 residues involved in H-bond interactions; **(D)** Interatomic H-bond distances verified over 20 ns of MD simulation of TFL1 structure.

Two important structural features of the FT protein regarding the regulation of flowering include the surface-exposed loop region, named segment B (residues 128–141) encoded by the fourth exon which is involved in PC binding, and Tyr85, a key functional residue that differentiates FT activity from the floral repressor TFL1 (Lee et al., 2013; Zhang et al., 2015). Structural analyses of the PC binding site of the FT structure revealed that the residue Tyr85 formed an H-bond with the oxygen of residues Glu109 and Gln112, which confers stability to the PC binding site. Similarly, residue Gln140, located in segment B is also involved with the H-bond network, whereas His87 and Arg139 stabilize the spatial coordination of Tyr85 by van der Waals interaction (Nakamura et al., 2019). Moreover, studies have also demonstrated the formation of H-bonds between the residue His88 with Asp144 in the TFL1 structure of *A. thaliana* (Ho and Weigel, 2014). Based on these assumptions, we performed a structural analysis of H-bond interactions in the adjacent residues to Tyr85 in FT and His88 in TFL1 structures through the MD simulation in the ancestral sequence representative to angiosperms clade of both proteins. Our analyses demonstrate that these interactions maintain stability over the MD simulation (Figures 7A–D), thus indicating the conservation of these residue interactions over evolutionary time.

Our structural analysis showed that the FT Tyr85 residue (Tyr84 in the ancestral sequence from angiosperms) does not form an H-bond, however, Glu109 (Glu108 in angiosperms) does interact with Arg111 (Figure 7A). In the structure of TFL1, Asp144 (Asp139 in the ancestral sequence of angiosperms) interacts with the residues Ser137 and His140. Differently, His88 (His84 in the ancestral sequence of angiosperms) does not interact with the other adjacent residues (Figure 7C).

### Analysis of mutations occuring during F/TFL1 evolution and their effects on protein structural stability

**FIGURE 8:**
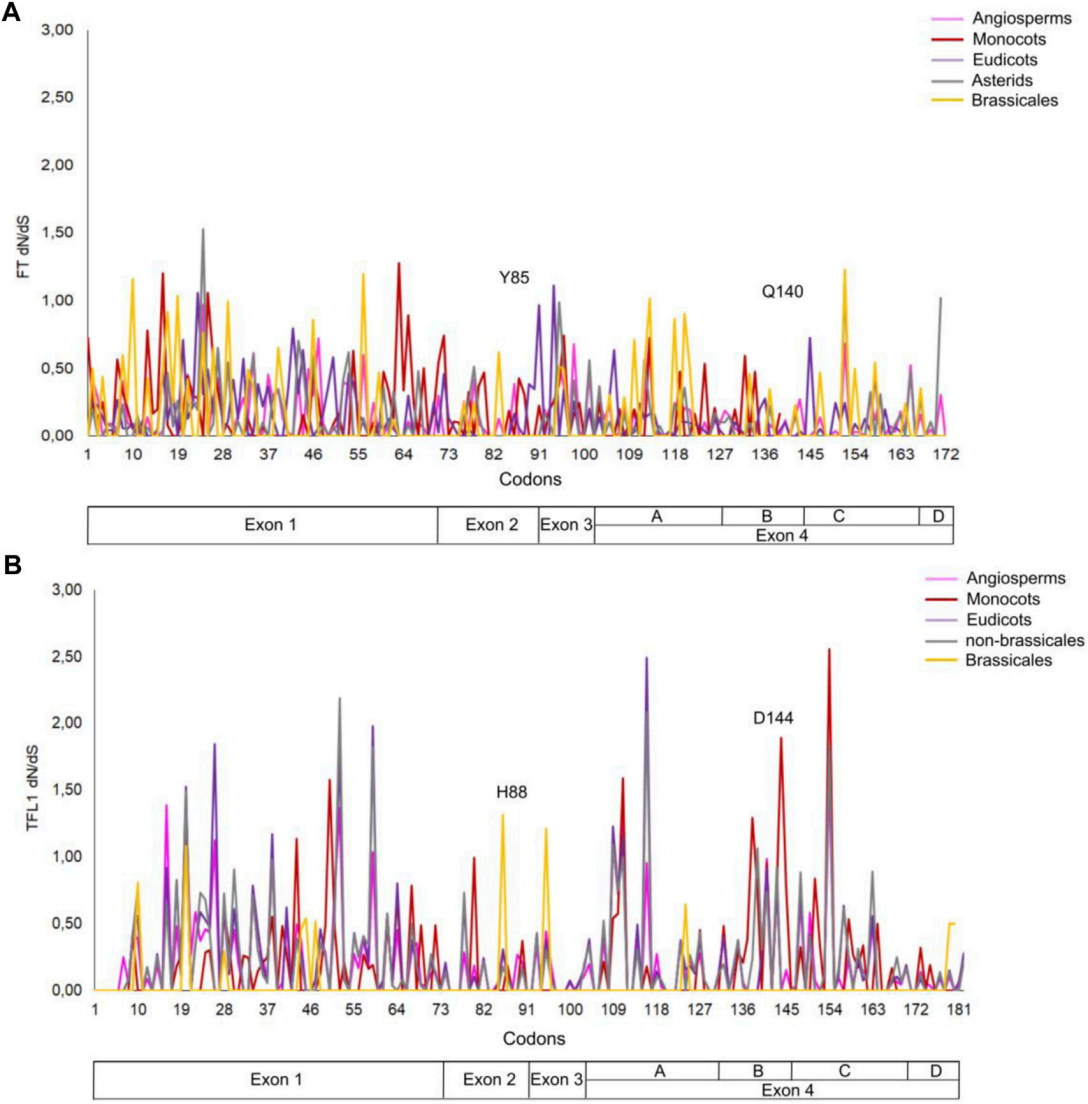
Analysis of the selective pressures indicated by dN/dS rates of the codons for FT **(A)** and TFL1 genes **(B)**.

The number of mutations that confer advantageous changes during the evolutionary process is limited due to the crossing of an energetic barrier of the fitness landscape of protein structures that could lead to alterations in their stability and function (Tokuriki and Tawfik, 2009; Faber et al., 2019). Mutational analysis has been widely applied to correlate the structure with protein function (Brandt et al., 2014; da Costa et al., 2017; Bhattacharya et al., 2018), organism phenotype (Neves Cruz et al., 2019), and to analyze protein evolution from ancestral sequences and adaptative evolutionary constraints in proteins structures (Studer et al., 2014a; Sharir-Ivry and Xia, 2018). In the present study, we performed a mutational analysis for FT and TFL1 structures using alanine scanning to investigate the influences of mutations in the structural stability in both proteins and correlate these results with the selective pressures indicated by dN/dS rates of the FT (Figure 8, panel A) and TFL1 (Figure 8B) codons.

**FIGURE 9:**
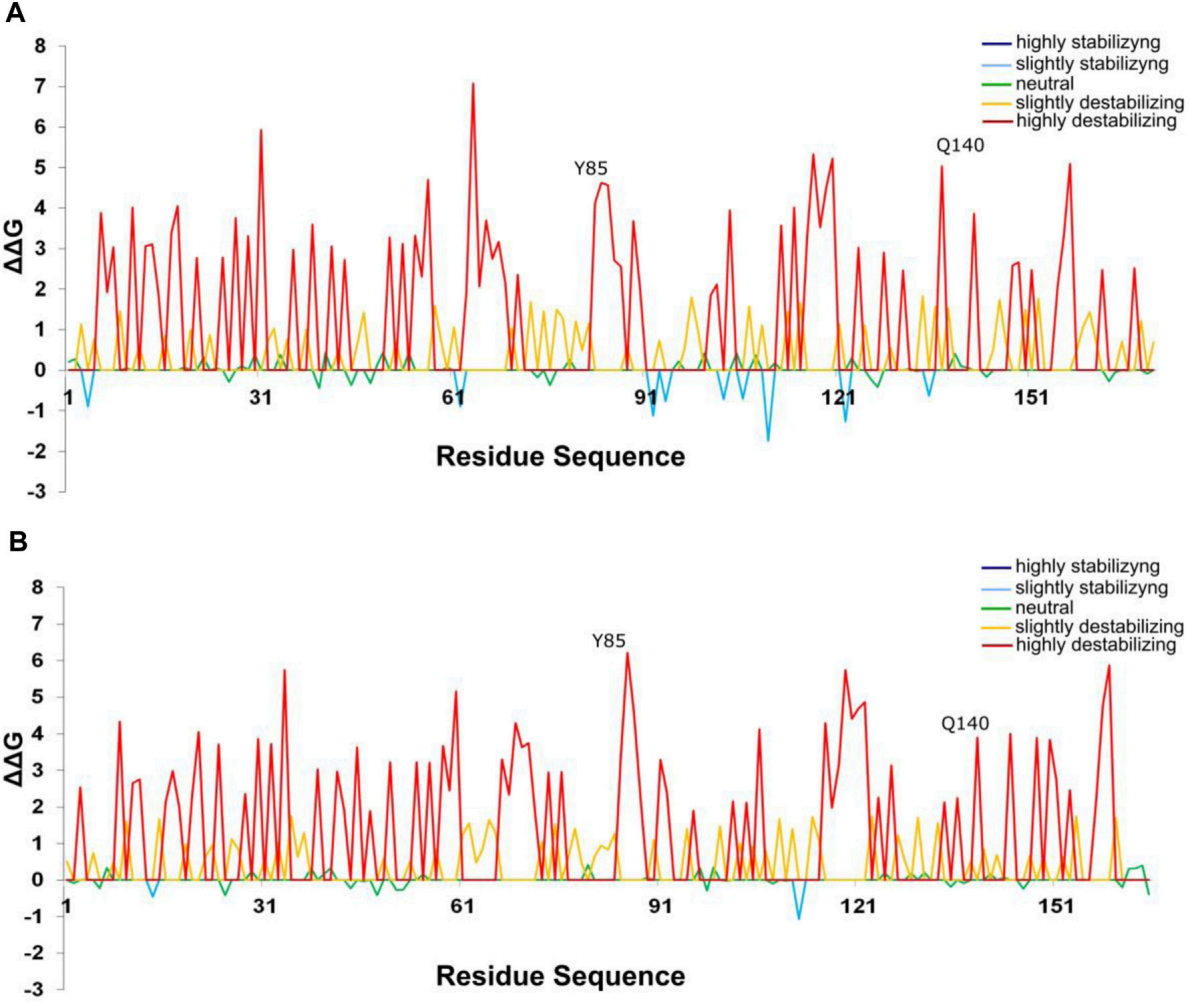
Most alanine substitutions in the FT structure are destabilizing, especially at the residue Tyr85. Alanine scanning plot of FT structures showing the effect of mutations on protein stability **(A)** Inferred ancestral protein sequence of angiosperms. **(B)** Protein sequence of *A. thaliana.*

Our results reveal that most alanine substitutions in FT and TFL1 sequences are destabilizing, especially at residue Tyr85 that plays an important role in FT activity, and at residue His88 in TFL1 structure that is involved with the flowering repression (Ahn et al., 2006a; Ho and Weigel, 2014). The residues from the P-loop domain (Pin and Nilsson, 2012) of FT structure from *A. thaliana* showed predominantly high destabilizing mutations (Figure 9), especially the residues Phe128 (+4.86 kcal mol^−1^), Gln130 (+2.25 kcal mol^−1^), Gly132 (+3.12 kcal mol^−1^), Gly140 (+2.12 kcal mol^−1^), Arg142 (+2.24 kcal mol^−1^), and Phe145 (+3.89 kcal mol^−1^) (Figure 9). Moreover, we found that the codons that encode these residues are under constant selective pressure, thus corroborating with previous findings for the *FT* gene (Klintenäs et al., 2012b; Mackenzie et al., 2019b). In contrast, *TFL1* demonstrated a predominant neutral natural selection (Figure 8).

Analyzing dN/dS rates of the residues involved with the H-bond interactions in the FT structure present in *A. thaliana* and ancestral sequence of the angiosperm clade, we observed the presence of negative pressure (dN/dS<1), and the presence of destabilizing mutations (ΔΔG>0), suggesting purifying selection over evolutionary time. These findings also corroborate the previous observation that the presence of H-bond interactions in the PC binding site maintains its structural stability (Nakamura et al., 2019). Similarly, mutations in the residues that formed H-bond interactions in the PC binding site were destabilizing for overall protein structure and showed purifying selection in ancestral angiosperm and *A. thaliana* TFL1.

We also performed analyses of the evolutionary rates of the ancestral proteins of each clade based on the inferred phylogenies (Figures 1, 2). Considering the *FT* sequences, we observe that the angiosperm clade showed a low dN/dS rate when compared with the other ancestral sequences (ω_angio_ = 0.065). The class of substitution sites selected were the following: neutral (ω = 1), sites under positive selection (ω > 1), and sites under negative selection (ω < 1) (Nei and Gojobori, 1986). The ancestral sequences of *FT* probably evolved under negative selection with low rates or absence of non-synonymous substitutions, which conserved the protein structure during the evolutionary history that led to the appearance of flowering plants. The ancestral sequences of monocots exhibited a dN/dS rate ω = 0.081, which suggests that the occurrence of synonymous mutations was influenced by natural selection (negative pressure). There is evidence that natural selection is purifying, which may explain little change in ancestral sequences. Similar results were also found for ancestral sequences of eudicots (ω = 0.087) and asterids (ω = 0.078). In contrast, the ancestral sequence of brassicales exhibited a high dN/dS rate (ω = 0.147) when compared with the other analyzed sequences which could indicate the predominance of non-synonymous mutations at the residues sites. The negative pressure of the *FT* gene (rates dS > dN) could be related to its different molecular functions in the flowering process (Pin and Nilsson, 2012). Analyzing the evolutive trajectory of *FT* in the monocots, we noticed that majority of the investigated species showed dN/dS rates compatible with purifying selection, similarly when compared with the ω value for the clade (ω = 0.081), thus showing few residues under positive selection (Supplementary Information S9).

Regarding the ancestral *TFL1* sequences of the angiosperm’s clade, we obtained a ω = 0.115, and for monocots ω = 0.186, eudicots ω = 0.247, non-brassicales ω = 0.300 and brassicales ω = 0.236 (Figure 2). As previously discussed, the key residues His88 and Asp144 involved with TFL1 function remained under negative selection, however, we document regions of exon 4 under positive selection (angiosperms with residues Val109, Ser111, Lys154; monocots: Gly111, Gln138, Ala144, Gln154; eudicots: Val109, Ser111, Arg116, Lis154; non-brassicales: Val109, Arg116, Tre139, Lis154) (Supplementary Information S10, S11). This is reflected in the increase in dN/dS over time when compared to the values found for FT, which may indicate an adaptive modification of TFL1 protein structure (Benner et al., 2002), possibly due to its influences on different phenotypic traces of the flowering plants (Rallapalli et al., 2014). Additionally, TFL1 proteins probably evolved by a stability regime with a balance between stabilizing and destabilizing mutations that could lead to the divergence of large groups in the angiosperms.

During the evolutionary process, new mutations can be retained in the genomic pool by the relative strengths of natural selection and genetic drift. Regarding the natural selection forces, the rate of fixation of these mutations is accelerated by positive selection, under which favorable mutations to protein function or stability tend to be retained and in contrast, it is decelerated by the negative selection that tends to remove from the genomic pool disadvantageous mutations (Wang et al., 2019). Moreover, different studies (Sen et al., 2011; Studer et al., 2014b; Piot et al., 2017) have demonstrated that the appearance of destabilizing mutations in residues directly involved in the active sites could be selected by positive selection, and that such mutations may be functionally necessary as they may contribute to increasing the conformational flexibility of some regions and allowing adaptation under different conditions, which may be the case of amino acid changes in the 4th exon region of the TFL1 protein in *A. thaliana*. In addition, this destabilization can be compensated for by the occurrence of stabilizing mutations in other sites such as the formation of a new H-bond between amino acids Asp139 and Ser137 (Figure 7B) that contributes to maintaining the overall protein stability (Buller and Townsend, 2013; Studer et al., 2014a; Sharir-Ivry and Xia, 2018). Indeed, our analysis of the evolutionary rates for codons regions of the 2^nd^ and 4^th^ exons of FT gene that encode the P-loop domain and PC binding site found dN/dS < 1, and for TFL1 some codons that encode residues from the 4^th^ exon region found values equal to dN/dS = 1 and dN/dS > 1.

## Conclusion

Here, we have reconstructed the evolutionary history of FT/TFL1-like proteins in the main flowering plant groups and by combining ancestral sequence inference with structural and mutational analyses, we have identified the main residue sites that evolved by evolutionary constraints, altered the protein stability, and inverted their function from activation or repression of flowering time. Our results show that the main sites are conserved under negative selection, which includes the P-loop domain and PC binding site of FT structures. The residue Tyr85 located at the PC binding site of FT structure forms an H-bond with the oxygen of the residues Glu109 and Gln112 and confers stability to protein structure. Similarly, the residue Gln140, located in segment B also forms H-bond interactions, whereas His87 and Arg139 stabilize the spatial coordination of Tyr85*.* The presence of destabilizing mutations and negative selective pressures in residues located at the phosphatidylcholine binding site involved with H-bond formation indicate their structural role to maintain FT overall stability throughout evolution. In addition, residues of the 4^th^ exon are found to be under positive selection and they may be involved in the conformational alterations of proteins. However, the formation of H-bonds that confer stability to the structure may be indicative that structural changes are compensated by the occurrence of stabilizing mutations to maintain the overall stability of the protein. Finally, our study opens up new insights to understand the roles of natural selection in the adaptative evolution of flowering proteins in angiosperms and could further help crop improvement with an economic interest in flower and fruit industries.

## Data Availability

The datasets presented in this study can be found in online repositories. The names of the repository/repositories and accession number(s) can be found in the article/Supplementary Material.
