# Supplementary material for: Structural changes and adaptative evolutionary constraints in FLOWERING LOCUS T and TERMINAL FLOWER1-like genes of flowering plants
Source: Front Genet. 2022 Sep 29;13:954015. doi: 10.3389/fgene.2022.954015 (PMC9556947; doi:10.3389/fgene.2022.954015)
Supplement: Supplementary file 1 [file DataSheet1.docx]

Structural changes and adaptative evolutionary constraints in *FLOWERING LOCUS T* and *TERMINAL FLOWER1*-like genes of flowering plants

**SUPPORTING INFORMATION**

**Supporting information 01** –Sequence accession codes of *FT* and *TFL1* homologs of angiosperms and gymnosperms used to infer the phylogenetic trees. Each sequence is identified by its respective accession code retrieved from the GenBank database.

**Accession codes of *FT* sequences**

KU319433.1 *Eriobotrya deflexa* – Rosales

AB457620.1 *Ficus carica* FcFT – Rosales

KR872844.1 *Ziziphus jujub*a – Rosales

AB562502.1 *Gypsophila paniculata* – Caryophyllales

HM448912.1 *Beta vulgaris* – Caryophyllales

HM120863.1 *Cymbidium goeringii* – Asparagales

KU821026.1 *Hemerocallis middendorffii* – Asparagales

KF881011.1 *Dimocarpus longan* – Sapindales

JN214351.1 *Litchi chinensis* – Sapindales

AB671587.1 *Mangifera indica* – Sapindales

AB301934.1 *Citrus unshiu* – Sapindales

KF113360.1 *Paeonia suffruticosa* – Saxifragales

JQ686942.1 *Nicotiana tabacum* – Solanales

KC348396.1 *Solanum tuberosum* – Solanales

EF157728.1 *Vitis vinifera* – Vitales

HM631972.1 *Gossypium hirsutum* – Malvales

KY078855.1 *Shorea curtisii* – Malvales

AB706329.1 *Shorea beccariana* – Malvales

KY078857.1 *Shorea leprosula* – Malvales

KX346229.1 *Camellia oleifera* – Ericales

AB741571.1 *Camellia sinensis* – Ericales

JX417423.1 *Actinidia chinensis* – Ericales

AB775532.1 *Fagus crenata* – Fagales

JQ409561.1 *Betula platyphylla* – Fagales

JQ951966.1 *Betula luminifera* – Fagales

DQ865291.1 *Cucurbita maxima* – Cucurbitales

AB161107.2 *Populus nigra* – Malpighiales

AY515152.1 *Populu deltoides* – Malpighiales

XM 002316137.2 *Populus trichocarpa* – Malpighiales

HM030997.1 *Brassica oleracea* – Brassicales

KJ576855.1 *Boechera stricta* – Brassicales

AB465586.1 *Arabidopsis halleri* subsp *gemmifera* – Brassicales

NM 105222.3 *Arabidopsis thaliana* – Brassicales

AY705794.1 *Triticum aestivum* – Poales

HQ343233.1 *Ananas comosus* – Poales

JX141617.1 *Phyllostachys edulis* – Poales

KF853467.1 *Musa acuminata* – Zingiberales

LC030437.1 *Symplocarpus renifolius* – Alismatales

KM023154.1 *Persea americana* var *americana* – Laurales

HQ173333.1 *Aquilegia formosa* – Ranunculales

AB605176.1 Gentinales *Gentiana triflora* – Gentinales

JQ955602.1 *Coffea arabica* – Gentinales

JF434701.1 *Xanthium strumarium –* Asterales

AB602322.1 *Lactuca sativa* – Asterales

GQ884985.1 *Helianthus annuus* – Asterales

GU120195.2 *Chrysanthemum lavandulifolium* – Asterales

KM505151.1 *Punica granatum* – Myrtales

HQ538822.1 *Pisum sativum* – Fabales

HQ721813.1 *Medicago truncatula* – Fabales

EU287455.1 *Glycine max* – Fabales

JQ071503.1 *Arachis hypogaea* – Fabales

DQ865290.1 *Maxima* – Cucurbitales

EF462212.1 *Moschata* – Cucurbitales

KX270259.1 *Amentotaxus argotaenia* – Cupressales

KX270281.1 *Sciadopitys verticillata* – Cupressales

KX270277.1 *Podocarpus macrophyllus* – Araucariales

**Accession codes of *TFL1* sequences**

HQ173337.1 *Aquilegia formosa* – Ranunculales

NM 120465.3 *Arabidopsis thalian*a – Brassicales

JF436953.1 *Arabis alpina* – Brassicales

JQ071505.1 *Arachis hypogaea* – Fabales

HM641253.1 *Bambusa oldhamii* – Poales

AB017529.1 *Brassica rapa* – Brassicales

KC618321.1 *Cardamine flexuosa* – Brassicales

KX270264.1 *Cephalotaxus sinensis* – Pinidae

AB162044.1 *Chaenomeles sinensis* – Rosales

AB839767.1 *Chrysanthemum seticuspe* – Asterales

KT965640.1 *Cornus canadensis* – Cornales

KT965639.1 *Cornus florida* – Cornales

AB383153.1 *Cucumis sativus* – Cucurbitales

AB162043.1 *Cydonia oblonga* – Rosales

KC966719.1 *Dimocarpus longan* – Sapindales

AB162045.1 *Eriobotrya japonica* – Rosales

JF806631.1 *Fragaria vesca* – Rosales

FJ573236.1 *Glycine max* – Fabales

EU026442.1 *Gossypium* *arboreum* – Malvales

EU026443.1 *Gossypium* *hirsutum* – Malvales

EU026438.1 *Gossypium* *raimondii* – Malvales

KF581138.1 *Hemerocallis middendorffii* – Asparagales

KT279351.1 *Hevea brasiliensis* – Malpighiales

AB447465.1 *Hordeum vulgare* – Poales

AB219404.1 *Ipomoea nil* – Solanales

KJ130140.1 *Jatropha curcas* – Malpighiales

KC877999.1 *Litchi chinensis* – Sapindales

AF316419.1 *Lolium perenne* – Poales

AY423715.1 *Lotus japonicus* – Fabales

KM233713.1 *Oncidium hybrid* – Asparagales

KU561656.1 *Oxybasis rubra* – Caryophyllales

EF633467.1 *Picea abies* – Pinales

KX270294.1 *Picea wilsonii* – Pinales

KJ711113.1 *Pinus tabuliformis* – Pinales

AB104629.1 *Populus nigra* – Malpighiales

XM 002312775.1 *Populus trichocarpa* – Malpighiales

KM520125.1 *Punica granatum* – Myrtales

AB162048.1 *Pyrus communis* – Rosales

AB162041.1 *Pyrus pyrifolia* – Rosales

AB435524.1 *Raphanus sativus* – Brassicales

KJ496328.1 *Saccharum hybrid* – Poales

KY464932.1 *Tulipa gesneriana* – Liliales

AB588743.1 *Vigna unguiculata* – Fabales

GU947819.1 *Vitis aestivalis* – Vitales

GU947820.1 *Vitis californica* – Vitales

GU947821.1 *Vitis cinerea* – Vitales

GU947824.1 *Vitis mustangensis* – Vitales

GU947826.1 *Vitis riparia* – Vitales

DQ871591.1 *Vitis vinifera* – Vitales

DQ925416.1 *Zea mays* – Poales

**Supporting information 02** – Accession codes of the sequences and scientific names of the species used as fossil records for calibration to the most recent common ancestor.

| **tmrca** | **Age** | **Fossils** | **References** |
| --- | --- | --- | --- |
| Angiospermas FT/TFL1 | 136 Ma | *Amborella trichopoda* | Hughes & McDougall (1987); Hughes et al. (1991); Brenner (1996) |
| Monocotyledons FT/TFL1 | 112 Ma | *Liliacidites* sp. | Doyle & Hickey (1976); Doyle & Robbins (1977); Hickey & Doyle (1977); Doyle (1973); Walker &Walker (1984); Doyle et al. (2008) |
| Poaceae FT/TFL1 | 55.8 Ma | *Monoporites annulatus* | Adegoke et al. (1978); Muller (1981) |
| Eudicotyledons FT/TFL1 | 125 Ma | *Tricolpites micromurus* and *Hyrcantha decussata* | Hughes & McDougall (1990); Doyle et al.(1977); Doyle & Hotton (1991); Leng &Friis (2003, 2006); Dilcher et al. (2007) |
| Ericales FT/TFL1 | 89.3 Ma | *Pentapetalum trifasciculandricus* | Martínez-Millán et al. (2009) |
| Solanaceae FT | 33.9 Ma | *Solanispermum reniforme* and *Solanum arnense* | Chandler (1962); Martínez-Millán(2010) |
| Asteraceae FT | 47.6 Ma | *Raiguenrayun cura* and *Mutisiapollis telleriae* | Barreda et al. (2012) |
| Vitales TFL1 | 55.8 Ma | *Ampelocissus parvisemina* | Chen & Manchester (2007) |
| Sapindaceae FT | 55.8 Ma | *Aesculus hickeyi* | Hickey (1977); Manchester (2001) |
| Malvales FT/TFL1 | 55.8 Ma | *Malvaciphyllum macondicus* | Carvalho et al. (2011) |
| Brassicales FT/TFL1 | 89.3 Ma | *Dressiantha bicarpelata* | Gandolfo et al. (1998) |
| Brassicaceae FT | 23.03 Ma | *Thlaspi primaevum* | Becker (1961); Beilstein et al. (2010); Manchester &O’Leary (2010) |
| Fabales FT/TFL1 | 55.8 Ma | *Leguminocarpon gardneri* | Herendeen & Crane (1992) |
| Rosales TFL1 | 48.6 Ma | *Prunus wutuensis* | Li et al. (2011) |
| Fagales FT | 87.5 Ma | *Archaefagaceae futabensis* | Takahashi et al. (2008) |
| Cucurbitales FT | 48.6 Ma | *Cucurbitospermum sheppeyense* | Chandler (1961); Collinson (1983); Collinson et al. (1993) |
| Malpighiales FT/TFL1 | 89.3 Ma | *Paleoclusia chevalieri* | Crepet & Nixon (1998); Ruhfel et al. (2013) |


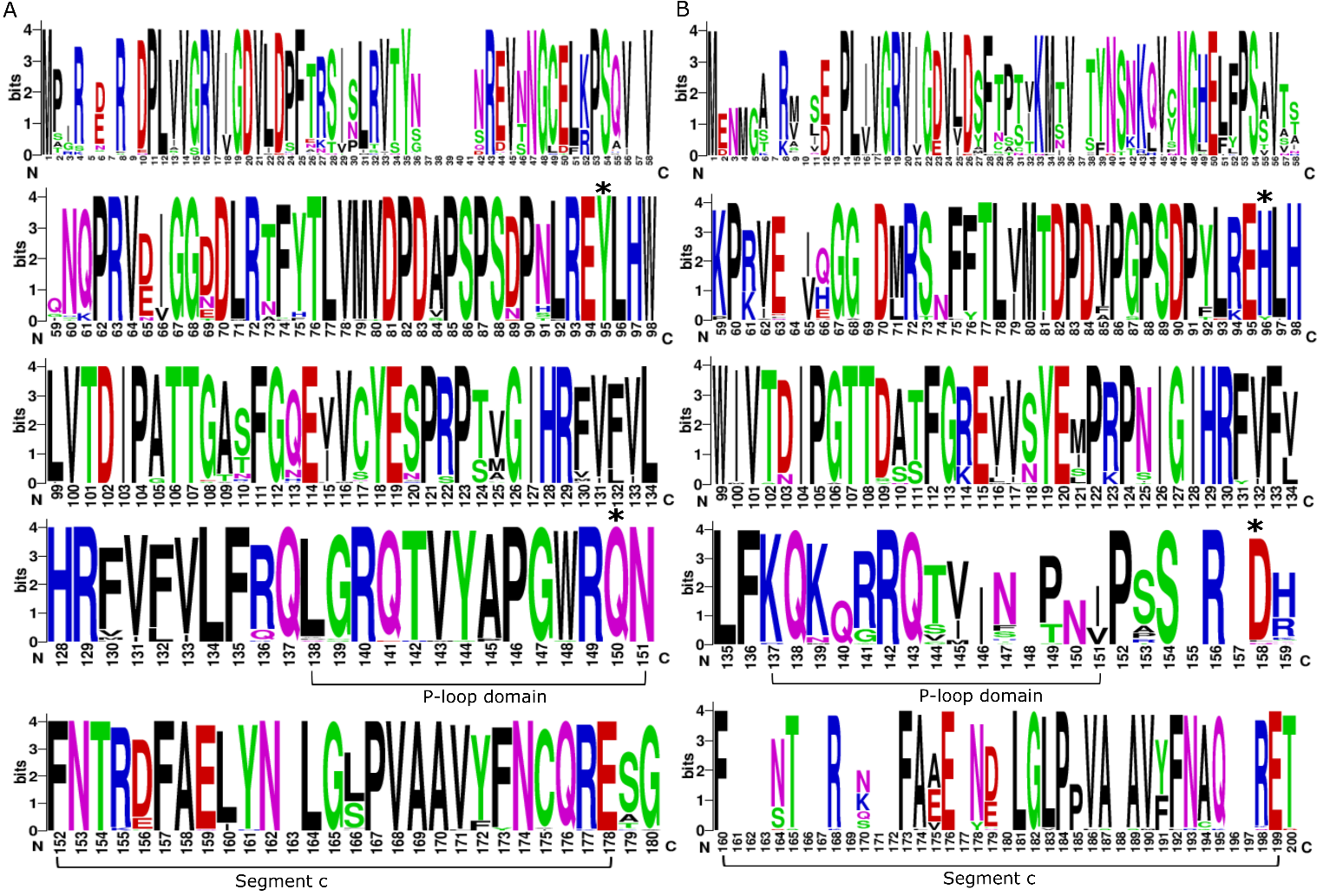


**Supporting information 03** – Schematic overview of ancestral residue sequences alignment showing the conserved regions of FT (panel A) and TFL1 (B). Key residues are highlighted by an asterisk.


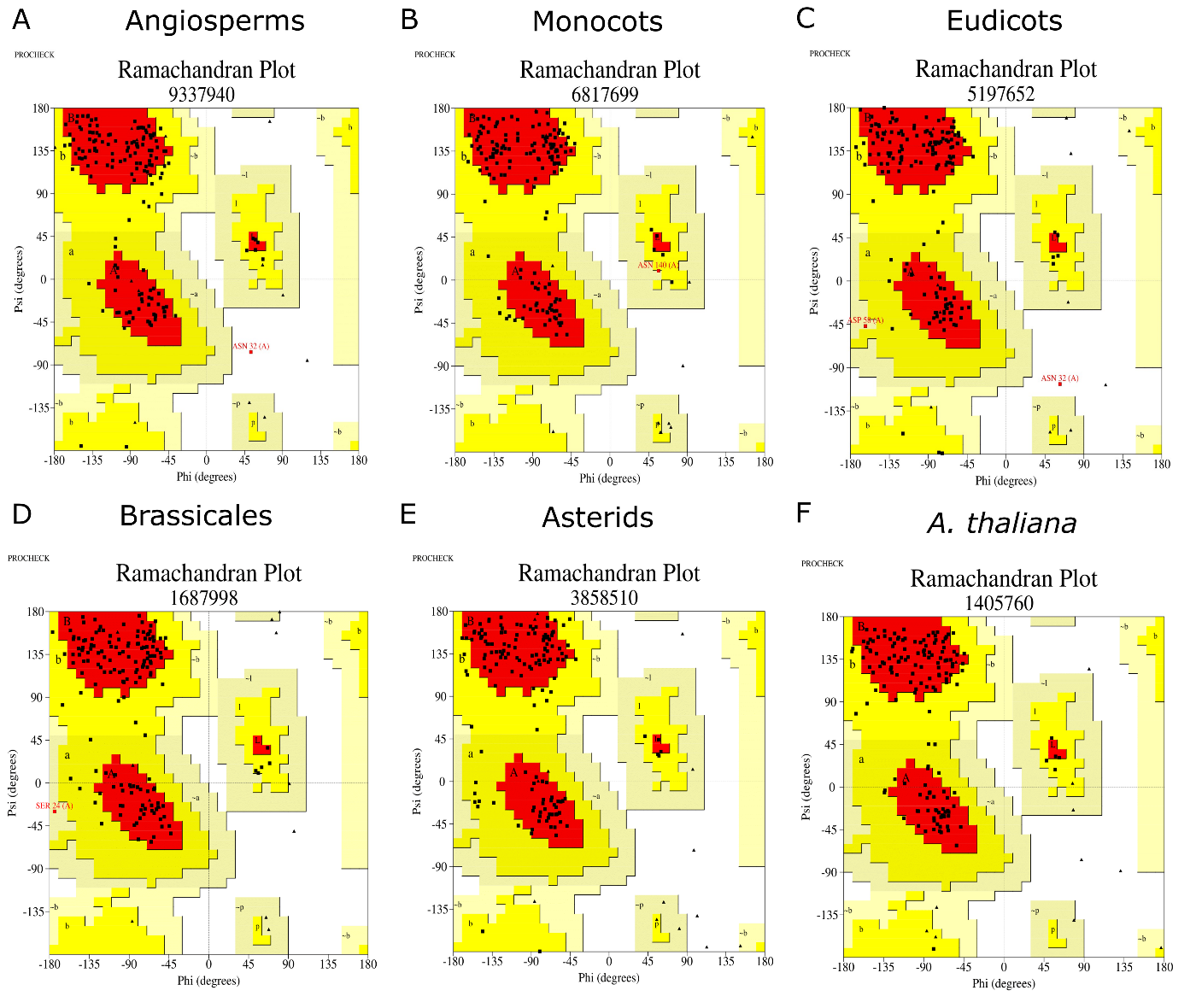


**Supporting information 04** – Ramachandran plots of the modeled structures of FT exhibited ≥ 79% of residues in the favorable regions (panels A to F). (A) angiosperms; (B) monocots; (C) eudicots; (D) brassicales (E) asterids, and (F) *A. thaliana*.


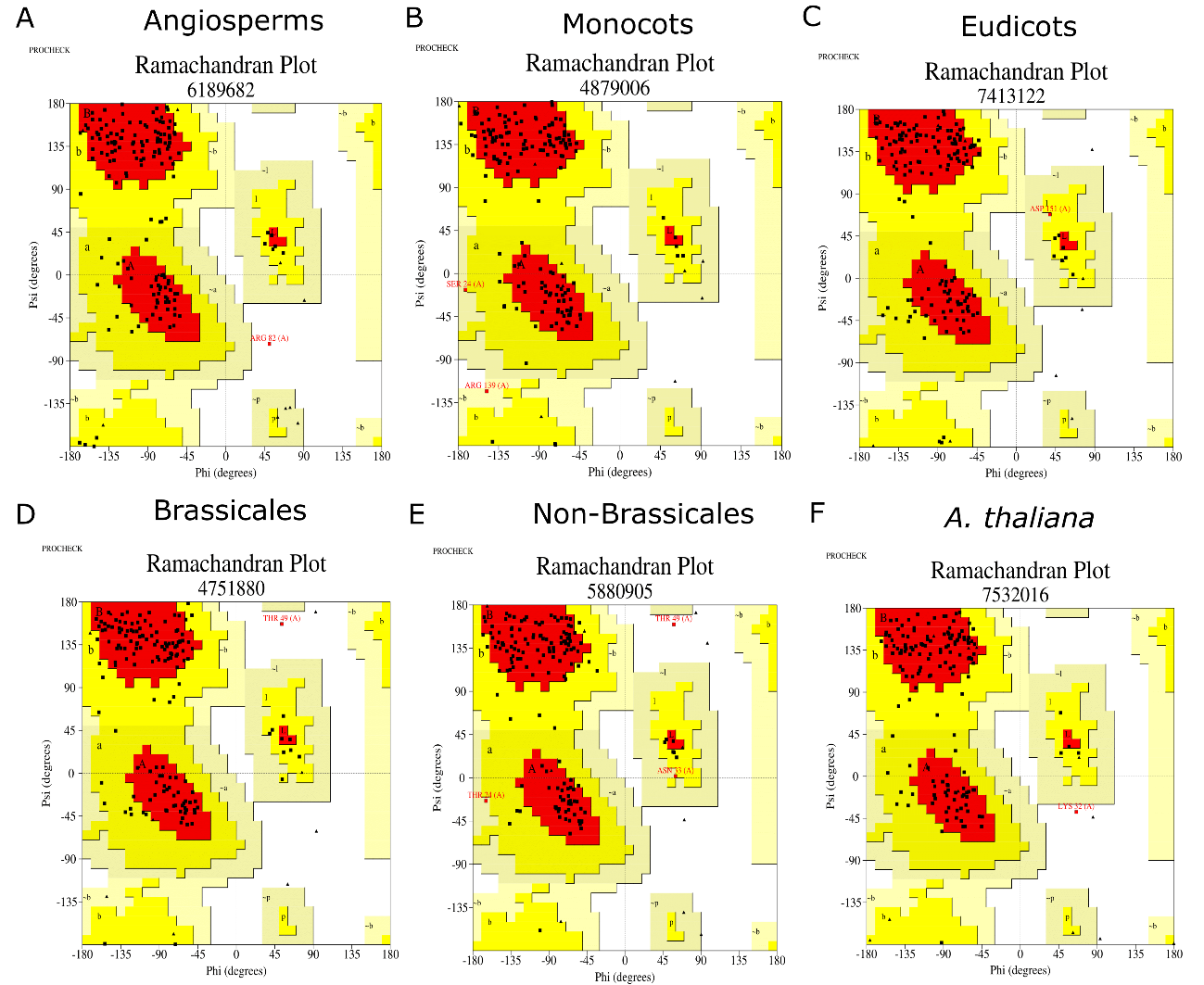


**Supporting information 05** – Ramachandran plots of the modeled structures of TFL1 exhibited ≥ 79% of residues in the favorable regions (panels A to F). (A) angiosperms; (B) monocots; (C) eudicots; (D) brassicales; (E) non-brassicales, and (F) *A. thaliana*.


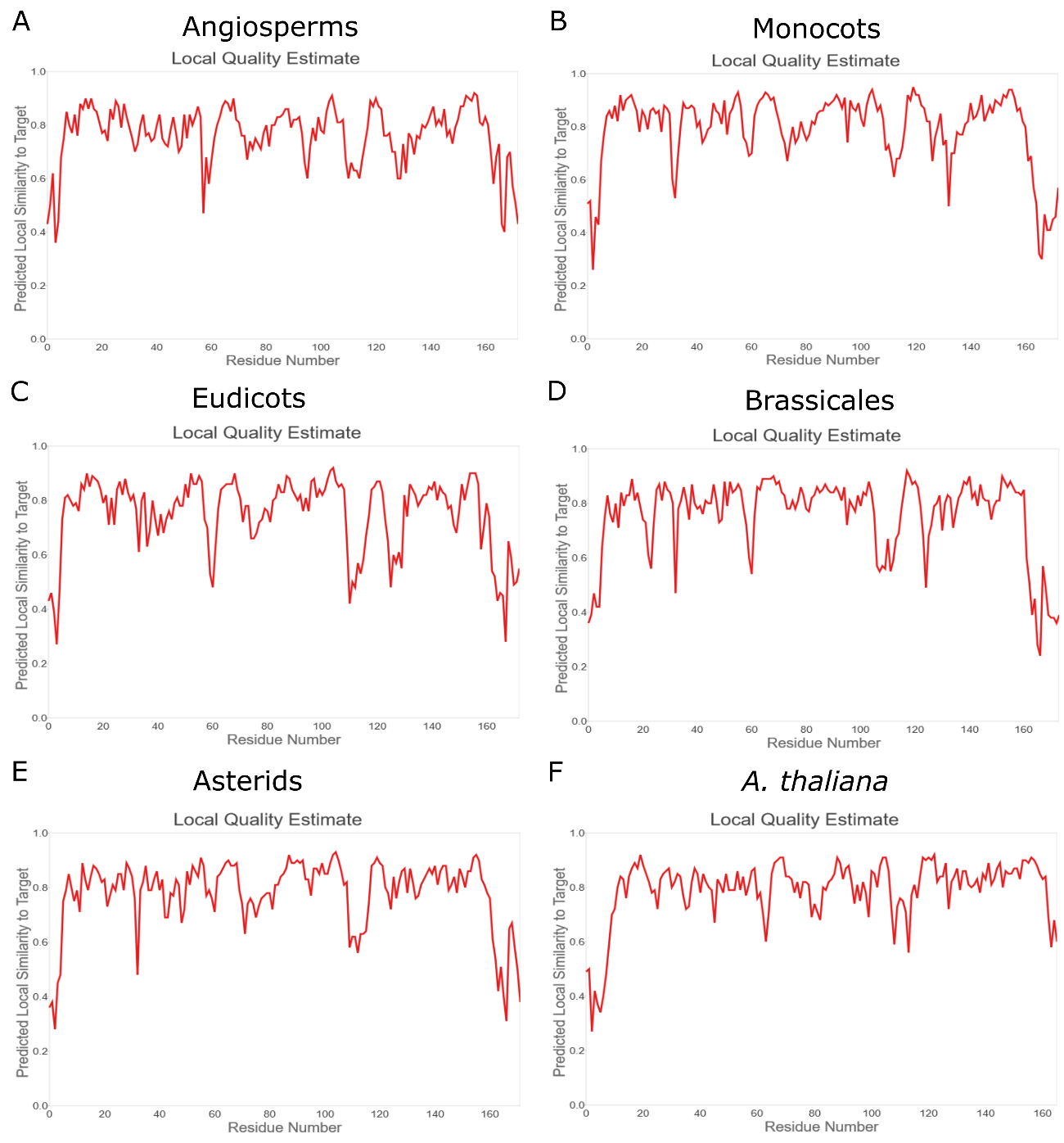


**Supporting information 06** – Local quality estimation exhibited by Qmean plot of the modeled ancestral structures of FT from angiosperms (panels A to E) and *A. thaliana* (F). (A) angiosperms; (B) monocots; (C) eudicots; (D) brassicales (E) asterids, and (F) *A. thaliana*.


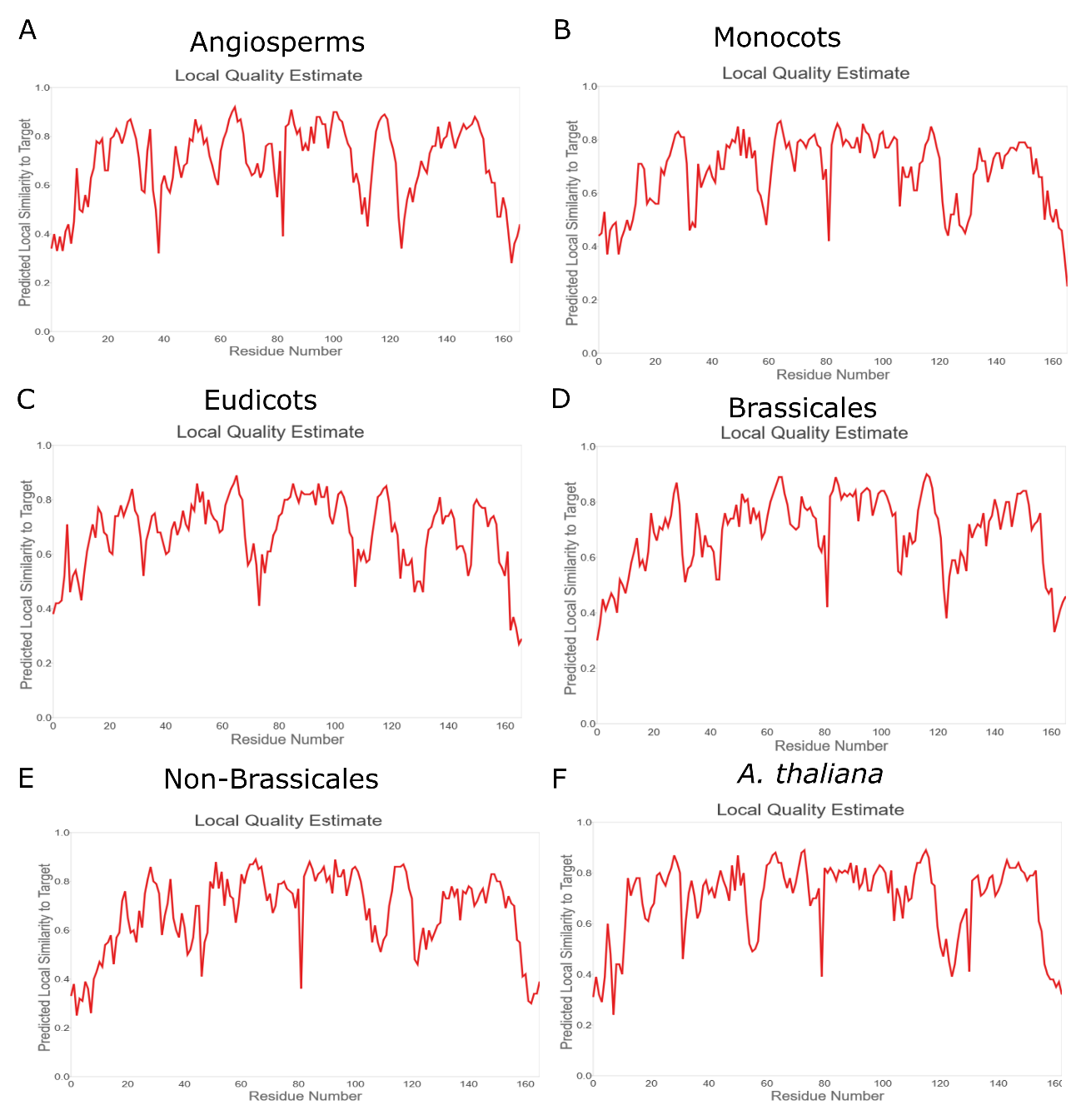


**Supporting information 07** – Local quality estimation exhibited by Qmean plot of the modeled ancestral structures of TFL1 from angiosperms (panels A to E) and *A. thaliana* (F). (A) angiosperms; (B) monocots; (C) eudicots; (D) brassicales; (E) non-brassicales, and (F) *A. thaliana*.


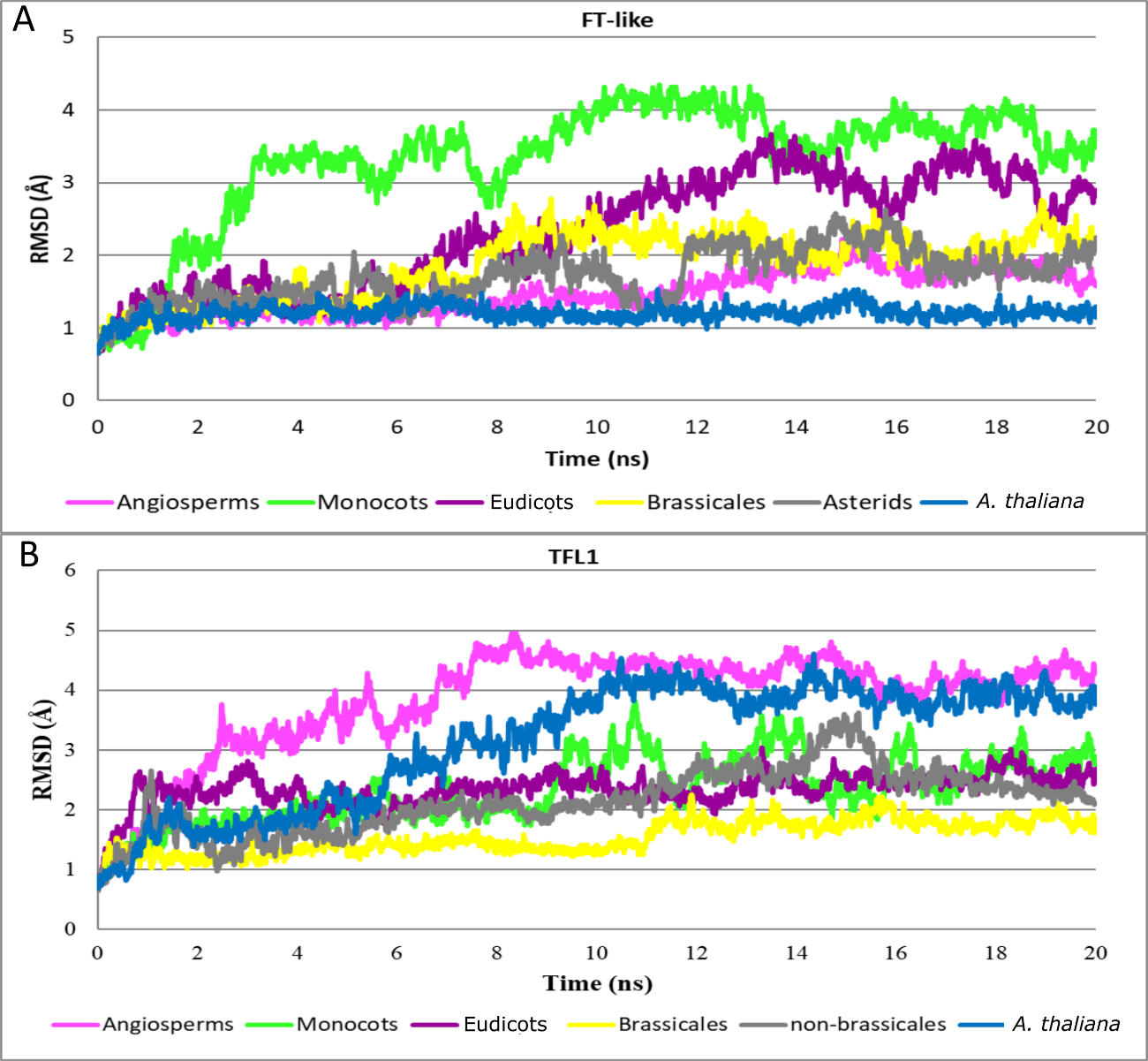


**Supporting information 08** – RMSD plots obtained over 20 ns of MD simulation for the modeled structures of FT (panel A) and TFL1 (B).


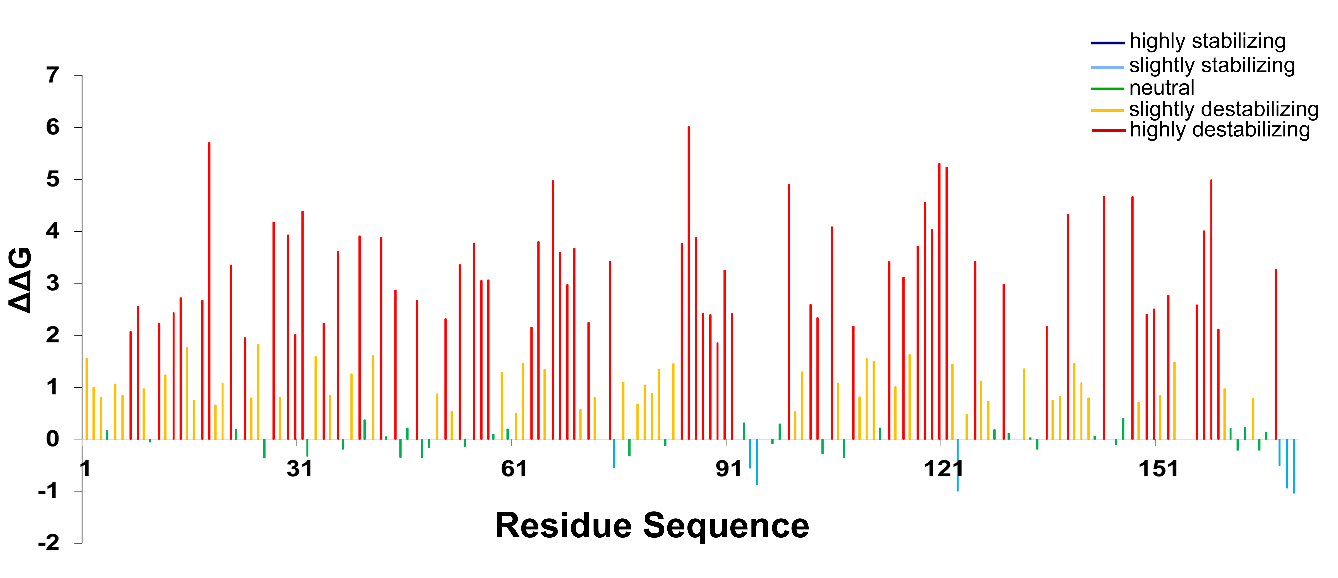


**Supporting information 09** – Alanine scanning plot of FT structures showing the effect of mutations on protein stability (A) brassicales


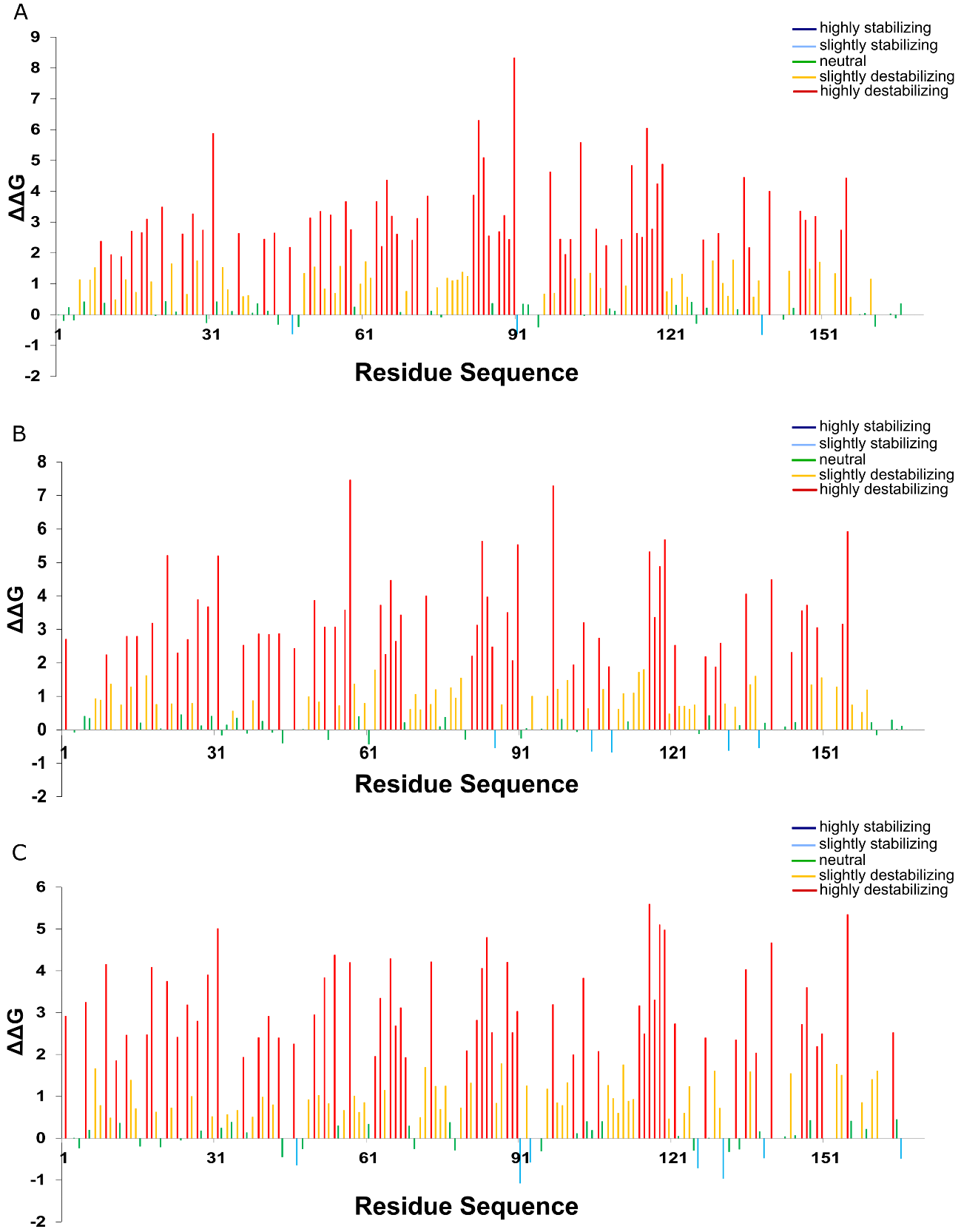


**Supporting information 10** – Alanine scanning plot of TFL1 structures showing the effect of mutations on protein stability (panels A to C). (A) angiosperms; (B) monocots and (C) eudicots.


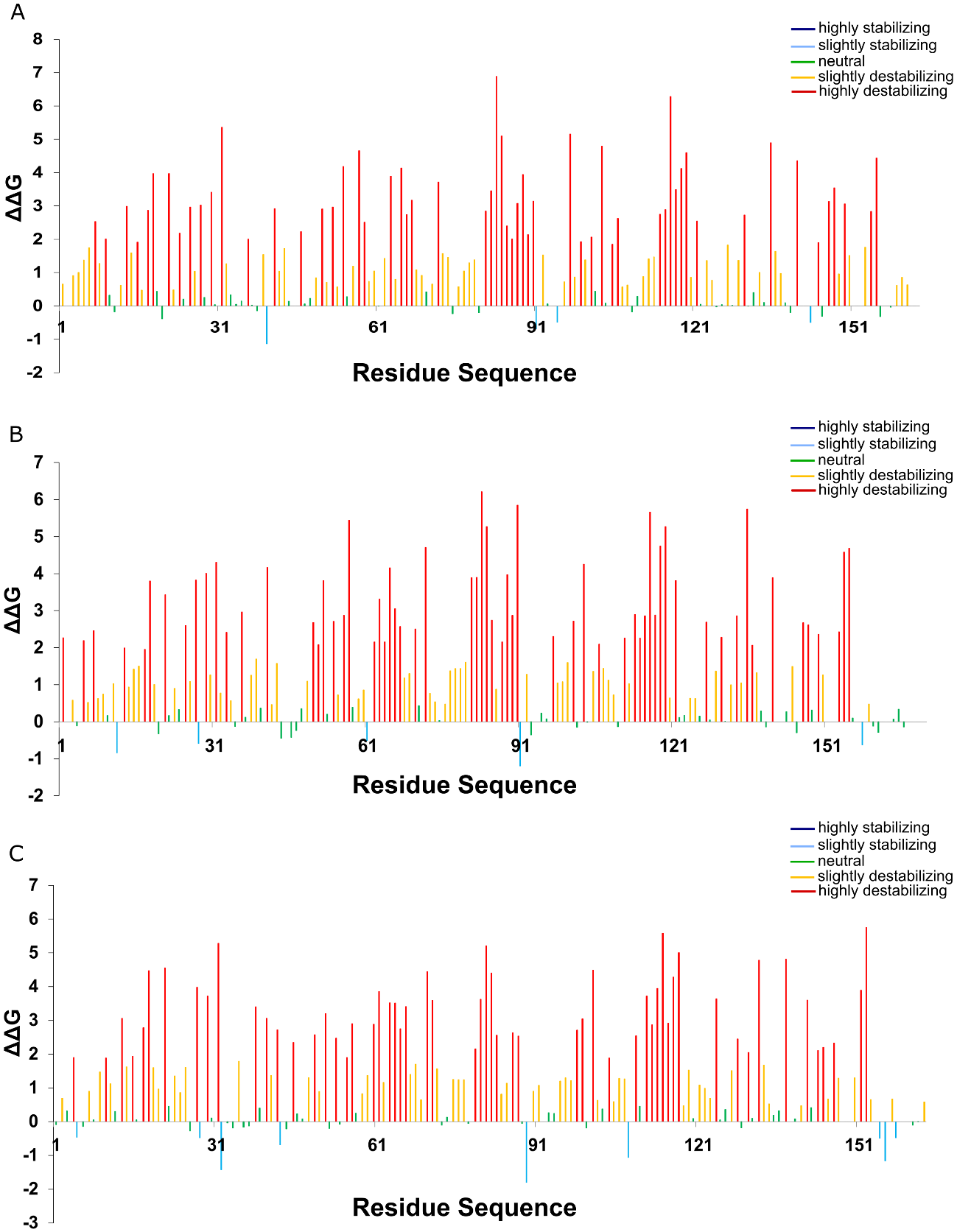


**Supporting information 11** – Alanine scanning plot of TFL1 structures showing the effect of mutations on protein stability (panels A to C). (A) brassicales (B) non-brassicales and (C) *A. thaliana.*
